# Systematical Analyses of Global Ionospheric Disturbance Current Systems Caused by Multiple Processes: Penetration Electric Fields, Solar Wind Pressure Impulses, Magnetospheric Substorms, and ULF Waves

**DOI:** 10.1029/2020JA027942

**Published:** 2020-08-27

**Authors:** Chao‐Song Huang

**Affiliations:** ^1^ Air Force Research Laboratory Space Vehicles Directorate Kirtland AFB NM USA

**Keywords:** current system, equivalent current, penetration electric field, solar wind pressure, magnetospheric substorm, ULF waves

## Abstract

We present the first systematic analysis of global ionospheric disturbance current systems caused by multiple processes of solar and magnetospheric origin, including reorientations of the interplanetary magnetic field (IMF), sudden changes in the solar wind dynamic pressure, magnetospheric sawtooth substorms, and ultralow frequency (ULF) waves. Measurements from global magnetometer networks are used to derive the equivalent disturbance currents from the polar cap to the equator. A surprising result is that the equivalent disturbance current systems are very similar, although the driving processes are completely different. The equivalent disturbance current system in response to IMF reorientation or substorm onset is characterized by a large vortex on the dayside and evening sector and a smaller vortex near dawn, and the polarity of the current vortices depends on the IMF direction. The equivalent disturbance current system caused by a sudden change in the solar wind pressure or by ULF waves consists of a single vortex at middle and low latitudes and a very small vortex above ~60° magnetic latitude near dawn. The similar disturbance current systems caused by different solar wind and magnetospheric processes suggest that the global distribution of the ionospheric currents is determined by the intrinsic property of the ionosphere. The global current system takes only ~1 min to completely reconstruct, indicating that the current system can reach a new steady state within 1 min. A scenario is proposed to explain the global distribution and fast reconstruction of the current systems.

## Key Points

- Global ionospheric equivalent disturbance current systems caused by multiple processes are derived and systematically analyzed for the first time
- A time scale of 1 min for global ionospheric disturbance current system to reconstruct is identified
- A scenario is proposed to explain the global distribution and fast reconstruction of the ionospheric current systems

## 1 Introduction

Many solar wind and magnetospheric processes can cause significant disturbances in the ionosphere. This study focuses on the processes that can generate nearly instantaneous response in equatorial ionospheric electric fields. A nearly instantaneous response means that when the solar wind or magnetospheric processes cause changes in electric fields in the polar ionosphere, corresponding or similar changes occur almost immediately in equatorial electric fields without obvious time delay. One such process is a southward turning of the interplanetary magnetic field (IMF) which causes the generation of penetration electric fields at low latitudes. Penetration electric fields are the electric fields of solar wind/magnetospheric origin observed equatorward of the shielding layer (Huang et al., 2006). The shielding layer, which is the inner edge of the plasma sheet, acts to shield regions equatorward (earthward) of it from the magnetospheric convection electric fields. Besides IMF southward turning, it has been observed that solar wind pressure impulses, magnetospheric substorms, and ultralow frequency (ULF) waves can also cause nearly instantaneous changes in equatorial ionospheric electric fields. This paper focuses on the four processes: reorientations of the IMF, sudden changes in the solar wind dynamic pressure, magnetospheric substorms, and ULF waves. The mechanism for transmission of the polar electric fields to the equatorial region during these four processes is believed to be the same. A brief review of each process is given below.

Penetration electric fields caused by IMF southward turning were first identified by Nishida (1968a, 1968b). It was found that quasiperiodic southward turnings of the IMF with a period of 30–60 min caused quasiperiodic enhancements in the northward component of the geomagnetic field measured by ground magnetometers near the magnetic equator. Because variations of the geomagnetic field are related to variations of ionospheric currents and electric fields, Nishida suggested that a southward turning of the IMF caused the generation of a zonal electric field in the equatorial ionosphere, and this IMF‐induced electric field is termed penetration electric field. Subsequent studies with measurements of ionospheric radars and satellites have verified that IMF southward turnings cause enhancements in equatorial vertical plasma drifts, corresponding to the occurrence of zonal penetration electric fields (Abdu, Jayachandran, et al., 1998, 2007; Fejer et al., 1979, 2007; Huang, 2008, 2019a, 2019b; Huang, Foster, et al., 2005, Huang et al., 2010, 2007; Kelley et al., 1979, 2003; Kikuchi et al., 1996; Tulasi Ram et al., 2012; Wei et al., 2008; Yizengaw et al., 2016). Equatorial vertical ion drifts associated with penetration electric fields are upward during the daytime, with a peak near 1900 LT, and downward in the midnight‐dawn sector (Fejer et al., 2008; Huang, 2015; Richmond et al., 2003). Penetration electric fields are quantitatively correlated with the interplanetary electric field, and the ratio of the penetration electric field in the dayside equatorial ionosphere to the dawn‐dusk component of the interplanetary electric field is 7–10% (Huang, 2019b; Huang et al., 2007; Kelley et al., 2003). The duration of penetration electric fields is controlled primarily by the duration of southward IMF and can be as long as 6 or even 14 hr (Huang, 2019b; Huang et al., 2010). Penetration electric fields have also been reproduced in numerical simulations (Maruyama et al., 2005, 2007; Richmond et al., 2003; Wang et al., 2008).

Sudden enhancements in the solar wind pressure cause a preliminary impulse in the geomagnetic field with a time scale of <1 min and sustained increase of the geomagnetic field over 5–10 min (Araki, 1977; Kikuchi & Araki, 1985; Kikuchi et al., 2001; Russell & Ginskey, 1995). Quasiperiodic oscillations in the solar wind pressure cause corresponding variations in the geomagnetic field (Motoba et al., 2003). Huang and Yumoto (2006) analyzed the quantitative correlation between the low‐latitude geomagnetic disturbances and solar wind pressure enhancements and found that the change of the low‐latitude geomagnetic field is linearly proportional to the change of the square root of the solar wind pressure. Huang et al. (2008) found that a sudden solar wind pressure enhancement causes an increase in the vertical plasma drift in the dayside equatorial ionosphere, and the increase in the equatorial vertical plasma drift, as well as in the geomagnetic field, exists for 30–40 min. They suggested that the increase in the equatorial vertical plasma drift is related to the occurrence of an eastward penetration electric field. In other words, a sudden enhancement in the solar wind pressure causes an eastward penetration electric field in the dayside equatorial ionosphere. Occurrence of upward plasma drifts or eastward penetration electric fields in the dayside equatorial ionosphere caused by sudden enhancements in the solar wind pressure has also been observed in other studies (Fejer & Emmert, 2003; Rout et al., 2016; Wei et al., 2012; Xiong et al., 2016).

Magnetospheric substorms also cause disturbances in electric and magnetic fields at low latitudes. Some studies showed that a negative magnetic bay, a decrease in the northward component of the geomagnetic field, occurred at middle and low latitudes during the expansion phase of substorms (Hashimoto et al., 2017; Hui et al., 2017; Kikuchi et al., 2000, 2001, 2003; Wei et al., 2009). Those studies suggested that substorm onset caused a westward electric field in the dayside equatorial ionosphere. At the dayside magnetic equator, the occurrence of a negative magnetic bay is attributed to a westward electric current, termed counter electrojet. Counter electrojet is related to a westward electric field because the dayside equatorial electrojet or counter electrojet primarily consists of the Pedersen current; the current and the electric field are in the same direction. However, it is important to note that the expansion onset of isolated substorms during magnetically quiet times is often triggered by a solar wind pressure impulse or by a northward turning of the IMF (McPherron et al., 1986). As discussed above, changes in the solar wind pressure or in the direction of the IMF also cause changes in equatorial electric fields. The occurrence of counter electrojet or westward electric fields at equatorial latitudes during substorm expansion phase may include, or even be dominated by, contributions from the solar wind pressure and IMF. Substorms can also occur during magnetic storm, and this type of substorms often shows a quasi‐periodicity of ~3 hr and is termed sawtooth events because energetic proton injection measured by geosynchronous satellites shows a sawtooth shape (e.g., Huang, 2002, Huang, Foster, et al., 2003; Huang, Reeves, et al., 2003, 2005). In order to single out the effects of substorm expansion phase on the equatorial ionosphere, Huang et al. (2004) and Huang (2009, 2012) analyzed the variations of low‐latitude electric and magnetic fields in response to substorm expansion onset during sawtooth events and found that the sawtooth substorm onset induces an eastward electric field and electrojet in the dayside equatorial ionosphere when the IMF remains continuously southward across the onset. Subsequent studies by Yamazaki and Kosch (2015) and Tulasi Ram et al. (2016) also found that the average electrojet perturbation at substorm onset is dominated by an eastward electric field. The studies of Huang (2004, 2009, 2012), Yamazaki and Kosch (2015), and Tulasi Ram et al. (2016) show that substorm onset, when the onset is not associated with a northward turning of the IMF, causes an eastward electric field, as well as an eastward electrojet, in the dayside equatorial ionosphere.

ULF waves that are excited in the magnetosphere can propagate along geomagnetic field lines to the ionosphere and cause corresponding disturbances in the ionosphere. There are numerous studies on ULF waves in the magnetosphere and in the high‐latitude ionosphere. The current study focuses on electric and magnetic field disturbances caused by ULF waves at middle and low latitudes. In particular, we will only analyze the waves at lowest frequency: Pc5 pulsations which have frequencies in the 2–7 mHz band (periods of 150–600 s). Magnetic field perturbations caused by Pc5 pulsations have been detected with ground magnetometers at low latitudes (Motoba et al., 2002; Reddy et al., 1994; Shinohara et al., 1998; Trivedi et al., 1997; Ziesolleck & Chamalaun, 1993). Ziesolleck and Chamalaun (1993) suggested that the spatial characteristics of Pc5 pulsations at low latitudes appear to be consistent with the magnetic ground signature of global compressional modes. Motoba et al. (2002) observed Pc5 magnetic fluctuations at low latitudes on the dayside and suggested that the dawn‐dusk electric field in the polar ionosphere accompanying a pair of field‐aligned currents (FACs) extends instantaneously to the equatorial ionosphere. Furthermore, Motoba et al. (2004) reported observations of HF Doppler oscillations in the low‐latitude ionosphere during a global Pc5 event and suggested that the HF Doppler oscillations arose from **E × B** effects due to direct penetration of a polar dawn‐dusk electric field.

The four processes, IMF reorientations, solar wind pressure changes, magnetospheric substorms, and Pc5 pulsations, all cause penetration electric fields at low latitudes. Increases in equatorial upward plasma drift, observed by radars and satellites, are considered to be direct evidence of penetration electric fields (Fejer et al., 1979, 2007, 2008; Huang, 2008, 2019a, 2019b; Huang, Foster, et al., 2005, 2007, 2008, 2010; Kelley et al., 1979, 2003; Wei et al., 2008); an increase in the upward plasma drift is related to an eastward penetration electric field. Because changes in electric field cause corresponding changes in ionospheric current, magnetic variations are also often used to characterize the occurrence of penetration electric fields. In fact, the earliest observations of penetration electric fields were based on magnetic field measurements (Nishida, 1968a, 1968b), and many subsequent studies also used magnetic field measurements to identify the signature of penetration electric fields (e.g., Kikuchi et al., 2000, 2001, 2003; Motoba et al., 2002, 2003, 2004; Yizengaw et al., 2016). Huang (2019a) compared variations of vertical ion drifts measured by the Jicamarca radar with variations of magnetic fields measured by ground magnetometers during penetration electric field events and found good agreement.

Magnetic variations are related to corresponding variations in ionospheric currents. The current system caused by IMF reorientations, solar wind pressure enhancements, or magnetospheric substorms extends from high latitudes to equatorial latitudes; this current system is termed the DP 2 current system (Kikuchi et al., 1996, 2000, 2001, 2003; Motoba et al., 2002, 2003, 2004; Nishida, 1968a, 1968b; Yizengaw et al., 2016). The global distribution of the ionospheric current system in association with penetration electric fields caused by IMF southward turning has been derived by Nishida (1968a, 1968b) and Huang (2019a); the current system is characterized by a large, counterclockwise vortex in the afternoon‐evening sector and a much smaller, clockwise vortex near dawn. Quasiperiodic oscillations in the IMF cause quasiperiodic variations in the DP 2 current system. The dayside ionospheric current distribution in response to solar wind pressure oscillations was derived at high latitudes in the Northern Hemisphere (>60° magnetic latitude, MLat) by Motoba et al. (2003), but a global picture of the currents at middle and low latitudes and on the nightside is not available. Global ionospheric current systems caused by magnetospheric substorms and Pc5 pulsations are not known.

This study aims at deriving global ionospheric disturbance current systems caused by multiple processes, including IMF reorientations, sudden changes in the solar wind pressure, magnetospheric substorms, and Pc5 pulsations. During magnetically quiet periods, the global ionospheric current system is termed the *S*_*q*_ current system and characterized by two large current vortices: a counterclockwise current vortex in the Northern Hemisphere and a clockwise current vortex in the Southern Hemisphere. The foci of the current vortices are located at middle latitudes near local noon (e.g., Yamazaki et al., 2009). A disturbance current system means that the background *S*_*q*_ current system is removed and only the disturbance currents are retained. We will compare the ionospheric disturbance current systems during the four processes and discuss the relationship among the disturbance current systems caused by different processes. We will also examine the time the ionospheric current system takes to reconstruct. On the basis of the observations, we will propose a scenario to explain the generation of the ionospheric disturbance current system in response to the solar wind and magnetospheric processes.

Another process that can cause large changes in equatorial electric fields is disturbance dynamo during magnetic storms. Enhanced Joule heating in the auroral zones during magnetic storms launches disturbance winds. When disturbance winds propagate to the equatorial region, they produce electric fields through the dynamo process. However, disturbance winds take, in general, several hours to travel from the auroral zones to the equatorial region, and equatorial electric fields generated by disturbance dynamo occur typically during the recovery phase of magnetic storms. Disturbance dynamo electric fields occur at equatorial latitudes several hours later than penetration electric fields (e.g., Huang et al., 2010) and are not discussed in this paper.

## 2 Observational Results

The ionospheric disturbance current system analyzed in this study is derived from global magnetometer networks. However, it is impossible to derive the true horizontal ionospheric currents only from ground magnetic perturbations because the horizontal currents, field‐aligned currents, distant currents in the magnetosphere, and even currents induced in the Earth's surface all contribute to the magnetic perturbations. Nishida (1968a, 1968b) and Kamide et al. (1976, 1981) used ground magnetometer data to construct an equivalent current system that is assumed to flow in a shell at ~100 km altitude and produce the ground magnetic perturbations. For the purpose of deriving a gross contour of the equivalent current system, the overhead current vector at each observatory is obtained by a clockwise rotation of the magnetic perturbation vector through 90°, and the strength of the current is assumed to be proportional to the magnitude of the magnetic perturbation. This method has been fairly successful in deriving the approximate pattern of the global current system. The same method has been used in subsequent studies to derive disturbance currents caused by solar wind pressure oscillations and IMF reorientations (e.g., Huang, 2019a; Motoba et al., 2003).

Magnetic field data used in this study are taken from the SuperMAG magnetometer networks (Gjerloev, 2012); the data have temporal resolutions of 1 min. In the SuperMAG data, the daily baseline is already removed, so the *S*_*q*_ current system is removed. As discussed in Huang (2019a), when we focus on the disturbance current system caused by short‐duration (from few minutes to ~1 hr) variations in the solar wind or magnetosphere, we can use a moving window to further remove the mean magnetic field. We will use the same or similar techniques in this study. The global disturbance current system is derived from measurements of 180–200 magnetometer stations in each case. Data from several magnetometer chains are used to show magnetic field variations along latitude and longitude. The stations of these latitudinal or longitudinal magnetometer chains are shown in the figures of this paper and listed in Table 1.

**Table 1:** Geographic and Geomagnetic Coordinates of Magnetometers

	Geomagnetic		Geographic	
Station	Longitude	Latitude	Longitude	Latitude
*Latitudinal chain in the African sector*				
Kiruna (KIR)	20.42	67.84	102.62	65.14
Hankasalmi (HAN)	26.65	62.3	104.78	59.17
Uppsala (UPS)	17.35	59.9	95.95	56.88
Hel (HLP)	18.82	54.61	95.32	50.93
LAquila (AQU)	13.32	42.38	87.68	35.74
Tamanrasset (TAM)	5.53	22.79	78.97	8.92
Addis Ababa (AAE)	38.77	9.03	111.51	−0.06
Bangui (BNG)	18.57	4.33	91.18	−9.54
Tsumeb (TSU)	17.7	−19.22	88.13	−30.50
Hermanus (HER)	19.23	−34.43	84.09	−42.08
Port Alfred (CZT)	51.87	−46.43	107.41	−53.23
*Latitudinal chain in the American sector*				
Postedela Baleine (PBQ)	282.26	55.28	0.2	65.01
Ottawa (OTT)	284.45	45.4	2.52	54.98
Fredericksburg (FRD)	282.63	38.2	−0.64	48.05
Bay St Louis (BSL)	270.37	30.35	−17.88	40.69
Jacksonville (JAX)	278.4	30.35	−7.11	40.78
Bullcreek (FIT)	279.05	28.07	−6.42	38.57
San Juan (SJG)	293.85	18.11	11.85	26.75
Huancayo (HUA)	284.67	−12.05	−2.75	1.17
Valdivia (VLD)	286.86	−39.48	−0.16	−25.61
Trelew (TRW)	294.68	−43.25	5.38	−29.98
Port Stanley (PST)	302.11	−51.7	11.06	−38.96
San Gregorio (ENP)	289.1	−52.13	3.05	−38.19
Puerto Natales (PNT)	289.1	−53.2	3.24	−39.23
Livingston Island (LIV)	299.61	−62.66	11.31	−48.75
Faraday Islands (AIA)	295.74	−65.25	9.72	−50.99
*Latitudinal chain in the Asian‐Australian sector*				
Magadan (MGD)	150.86	59.97	−139.32	54.3
Moshiri (MSR)	142.27	44.37	−145.03	37.64
Hatizyo (HTY)	139.8	33.12	−147.5	25.88
Chichijima (CBI)	142.3	27.15	−145.4	19.58
Guam (GUA)	144.87	13.59	−143.1	5.64
Biak (BIK)	136.05	−1.08	−151.37	−9.35
Charters Towers (CTA)	146.3	−20.1	−138.73	−28.92
Canberra (CNB)	150.7	−34.1	−131.11	−43.54
Dumont Durville (DRV)	140.01	−66.67	−122.99	−80.6
*Longitudinal Chain Close to the Equator*				
Bangui (BNG)	18.57	4.33	91.18	−9.54
Addis Ababa (AAE)	38.77	9.03	111.51	−0.06
Alibag (ABG)	72.87	18.62	146.22	11.99
Phuthuy (PHU)	105.95	21.03	178.77	14.11
Muntinlupa (MUT)	121.02	14.37	−166.45	6.9
Guam (GUA)	144.87	13.59	−143.1	5.64
Apia (API)	188.22	−13.8	−96.46	−15.5
Pamatai (PPT)	210.42	−17.57	−73.7	−16.22
Huancayo (HUA)	284.67	−12.05	−2.75	1.17
Mbour (MBO)	343.03	14.38	58.44	1.2

We analyze two or more cases for each of the four processes: IMF reorientations, sudden changes in the solar wind pressure, magnetospheric substorms, and Pc5 pulsations. IMF reorientations between southward and northward cause generation of penetration and shielding electric fields at middle and low latitudes. We choose two cases in which equatorial vertical plasma drifts by the Jicamarca radar are available so the occurrence of penetration electric fields can be justified. Sudden changes in the solar wind pressure actually include two subcategories: a sudden increase and a sudden drop. We choose two cases for each subcategory in which the change (increase or drop) in the solar wind pressure is large and occurs within a couple of minutes. For substorms, we choose the cases in which the onset of the substorm expansion phase occurs during continuous, stable southward IMF, so the possible effects of a northward turning of the IMF on the magnetic field and currents can be reduced to minimum. For Pc5 pulsations, we choose the cases in which the wave amplitude can reach 30 nT or so at low latitudes, so the increase or decrease of the magnetic field relative to its mean value can be unambiguously determined.

As already mentioned above, the quiet‐time *S*_*q*_ current system is removed in our analysis, and the derived current system is the disturbance current system. In the following sections, we use the term, “current system” but not “disturbance current system” in the text for simplicity. It should be remembered that the current system used in this paper is in fact a disturbance current system.

### 2.1 Current System Caused by IMF Reorientations

**Figure 1:**
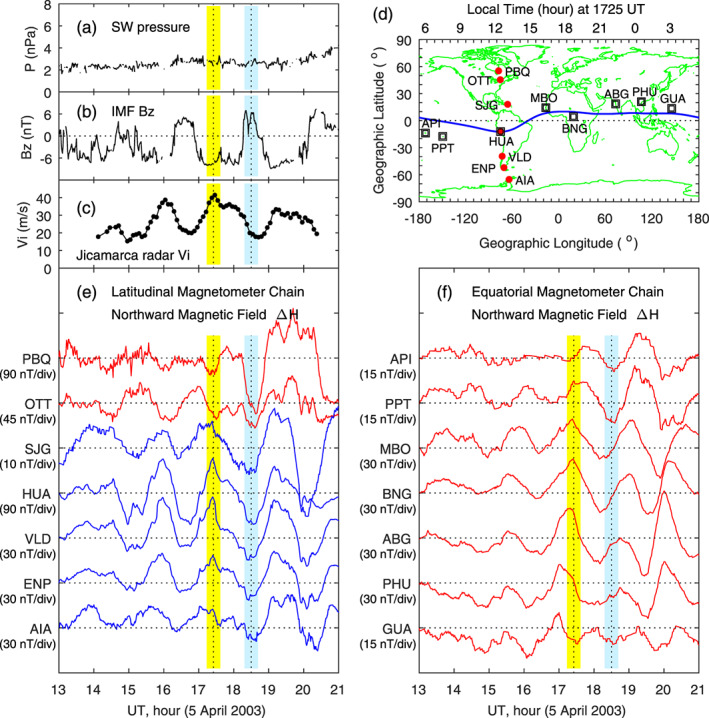
Geomagnetic variations associated with penetration and shielding electric fields caused by reorientations of the interplanetary magnetic field (IMF) on 5 April 2003. Shown in Figure 1 are (a) solar wind pressure, (b) IMF Bz, (c) equatorial vertical ion drifts measured by the Jicamarca radar, (d) magnetometer stations along the latitudinal chain and along the equatorial chain, (e) magnetic field northward component along the latitudinal magnetometer chain, and (f) magnetic field northward component along the equatorial magnetometer chain, respectively. The vertical yellow and blue bars denote the maximum and minimum in the equatorial vertical ion drift measured by the Jicamarca incoherence scatter radar. Local time (LT) at Jicamarca is universal time (UT)—5 hr. The scale of the magnetic field is given in the parenthesis underneath each station. The red and blue curves in Figure 1d are used to denote the phase reversal of magnetic perturbations along the magnetometer chain at 1725 UT.

Figure 1 shows the case of penetration electric fields on 5 April 2003. The solar wind dynamic pressure and IMF data plotted in Figures 1a and 1b, as well as in all other cases presented later, are taken from NASA Space Physics Data Facility (https://omniweb.gsfc.nasa.gov/) and have been shifted to the bow shock nose of the Earth. The solar wind pressure is mostly stable. The IMF *B*_*z*_ shows quasiperiodic (1.5–2 hr) oscillations between southward and northward. Plotted in Figure 1c is the equatorial *F* region vertical ion drift measured by the Jicamarca radar. The vertical ion drift is increased during southward IMF and decreased during northward IMF. This is a typical penetration electric field event.

Two magnetometer chains are shown in Figure 1d, and corresponding local times at 1725 UT are labeled on the top. The latitudinal magnetometer chain in the American sector, denoted by red dots, is located around 1200 LT at 1725 UT. The longitudinal magnetometer chain near the equator, denoted by open squares, covers different time sectors. The northward (*H*) component of the magnetic field data measured by these two magnetometer chains are depicted in Figures 1e and 1f. A 3‐hr moving window has been used to remove the mean value of the magnetic field at each station, the same method as that used by Huang (2019a), and the magnetic field variations are those caused primarily by the IMF reorientations. The two vertical dotted lines denote the peak and valley of the vertical ion drift and magnetic field perturbations at 1725 and 1830 UT, respectively. The IMF *B*_*z*_ is negative at 1725 UT, causing an increase in the vertical ion drift and northward magnetic field at low latitudes, corresponding to the occurrence of an eastward penetration electric field in the dayside equatorial ionosphere. At 1830 UT, the IMF *B*_*z*_ is positive, causing a decrease in the vertical ion drift and the northward magnetic field at low latitudes, corresponding to the occurrence of a westward shielding electric field. As discussed by Huang (2019a, 2019b), the westward electric field in the dayside equatorial ionosphere during northward IMF also includes the contribution from transmission of the reverse convection electric field in the polar cap. For simplicity, we will only use shielding electric field to describe the electric field caused by IMF northward turning and will not repeatedly mention the contribution from the polar reverse convection electric field.

There are interesting features that can be seen in the magnetic field data. The latitudinal magnetometer chain is near local noon at 1725 UT. The electric field over Jicamarca is eastward, and the northward magnetic field at middle and low latitudes along this magnetometer chain, plotted in Figure 1e, is increased. In contrast, the magnetic field at PBQ and OTT shows a decrease, opposite to the change of the magnetic field at lower latitudes. This difference is caused by the currents at different latitudes. As will be shown later, the disturbance current around 1725 UT is westward at PBQ and OTT, causing a decrease in the northward magnetic field, but becomes eastward at other latitudes, causing an increase in the northward magnetic field. In Figure 1f, the peak and valley of the northward magnetic field along the longitudinal magnetometer chain has an apparent shift. At 1725 UT, magnetometers at MBO, BNG, and ABG, as well as the latitudinal chain in Figure 1e, are on the dayside or in the evening sector, PHU and GUA are in the postmidnight sector, and PPT and API are near dawn. The northward magnetic field at MBO, BNG, and ABG on the dayside and evening sector reaches a peak, which is related to the occurrence of an eastward electric field/current. In contrast, the northward magnetic field at PHU, GUA, PPT, and API in the midnight‐dawn sector reaches a minimum, corresponding to the occurrence of a westward electric field/current. This variation of the electric field and current with local time is consistent with penetration electric fields (Fejer et al., 2008; Huang, 2015, 2019b; Richmond et al., 2003).

**Figure 2:**
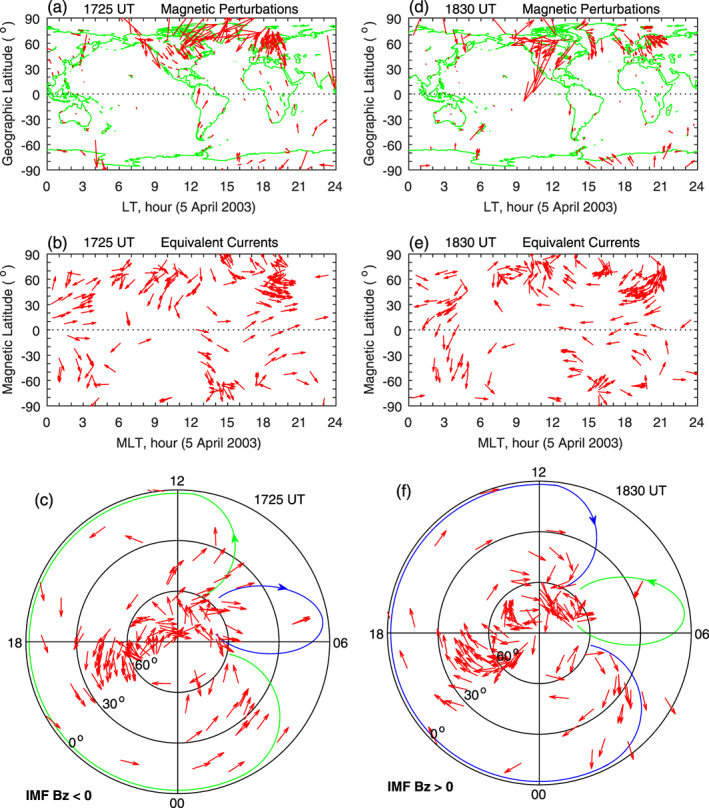
Global geomagnetic variations and ionospheric equivalent currents associated with penetration and shielding electric fields caused by southward and northward turnings of the IMF on 5 April 2003. Figures 2a–2c show global magnetic perturbations, global equivalent currents, and equivalent currents in the northern hemisphere at 1725 UT for southward IMF, respectively. Figures 2d–2f: Same as Figures 2a–2c but for northward IMF at 1830 UT.

As mentioned earlier, the daily baseline for any given station and any component in the magnetic field data has been removed (Huang, 2019a). Furthermore, the mean value of the magnetic field over a 3‐hr moving window is also removed. The magnetic perturbations, after the removal of the daily baseline and the 3‐hr mean value, are caused by the IMF reorientations. The left column of Figure 2 shows the magnetic field perturbations, the equivalent currents in magnetic local time (MLT)‐MLat coordinates, and the equivalent currents in the Northern Hemisphere in polar coordinates at 1725 UT. The magnetic field perturbations (Figure 2a) are large at high latitudes, indicating that the FACs flow into the high‐latitude ionosphere and cause global magnetic perturbations. The IMF is southward at this time, so the magnetic field perturbations and equivalent currents are related to penetration electric fields (e.g., Huang, 2019a).

Because the magnetic perturbations at middle and low latitudes and in the nighttime sector are much smaller than those at high latitudes, it is not possible to see the direction of the magnetic perturbations, as well as the equivalent currents, at the low‐latitude and nighttime stations. In order to show the direction of the equivalent currents at all stations, the current at each station is normalized to its amplitude, and this is the same as that used by Huang (2019a). The normalized currents at all stations have the same value (equal to one). The direction of the normalized equivalent currents at any station can be clearly seen. Throughout this paper, the equivalent currents are normalized in all cases. In Figure 2b, the current vector around 1200 MLT is directed southwest at 50–60° MLat in the Northern Hemisphere but becomes southeast at lower latitudes and in the Southern Hemisphere. Therefore, the northward magnetic field around 1725 UT is decreased at PBQ and OTT but increased at lower latitudes and in the Southern Hemisphere.

The global pattern of the equivalent currents can be better represented in polar coordinates. Figure 2c shows the equivalent currents in polar coordinates at 1725 UT. The current system is characterized by a large counterclockwise vortex covering the prenoon sector, the afternoon sector, and the evening‐midnight sector; the green curve with arrow is used to mark the outer contour of the counterclockwise current vortex. Near dawn, there is signature of a much smaller, clockwise vortex, although the structure of this clockwise vortex cannot be clearly identified because of sparse measurements there.

The magnetic perturbations and equivalent currents at 1830 UT during northward IMF are shown in the right column of Figure 2. The directions of the magnetic perturbations and equivalent currents during northward IMF in the right column are approximately opposite to those during southward IMF in the left column. The current system in Figure 2f is characterized by a large clockwise vortex covering the prenoon sector, the afternoon sector, and the evening‐midnight sector, as marked by the blue curve. This current system is related to shielding electric fields during northward IMF.

In the case of Figure 2, there is almost no magnetic measurement at middle and low latitudes near dawn, and the current distribution cannot be determined. In the studies of Nishida (1968a, 1968b), a relatively small current vortex exists near dawn, and the polarity of the dawn current vortex is opposite to that of the afternoon current vortex. We will also show that such a current vortex near dawn indeed exists when magnetometer measurements are available in other cases. Accordingly, we use the blue curve in Figure 2c and the green curve in Figure 2f to indicate the approximate location of the dawn current vortex.

Throughout this paper, a green curve with arrow is used to denote the outer contour of the counterclockwise current vortex, and a blue curve with arrow is used to denote the outer contour of the clockwise current vortex, as those depicted in Figures 2c and 2f. The green or blue contour can be in the dawn or afternoon sector, depending on the polarity of the current vortex. The green and blue curves will be used in all figures hereinafter and will not be repeatedly explained.

**Figure 3:**
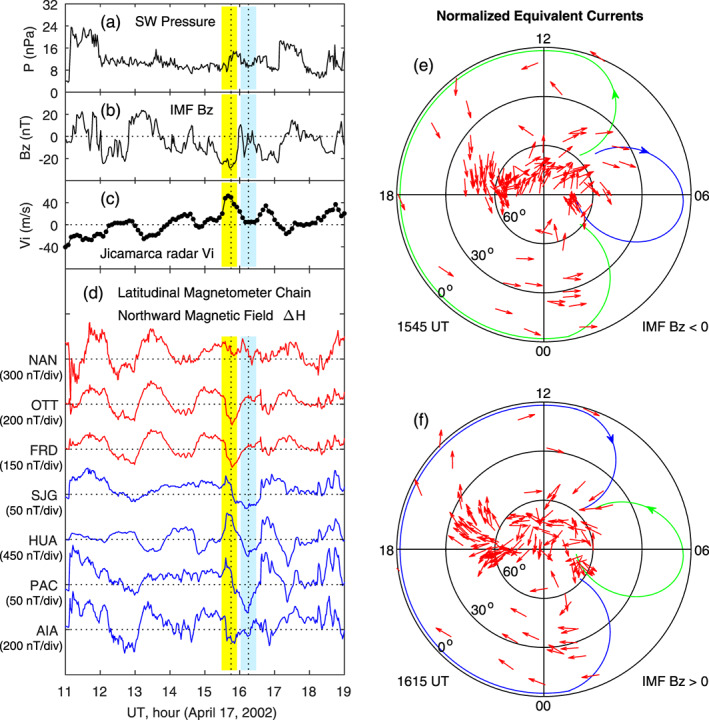
Geomagnetic variations and ionospheric equivalent currents associated with penetration and shielding electric fields caused by southward and northward turnings of the IMF on 17 April 2002. Shown in Figure 3 are (a) solar wind pressure, (b) IMF Bz, (c) equatorial vertical ion drifts measured by the Jicamarca radar, (d) magnetic field northward component along the latitudinal magnetometer chain, (e) equivalent currents in the northern hemisphere at 1545 UT for southward IMF, and (f) equivalent currents in the northern hemisphere at 1615 UT for northward IMF, respectively. The vertical yellow and blue bars denote the maximum and minimum in the magnetic field along the latitudinal magnetometer chain in the American sector. LT along this magnetometer chain is about UT − 5 hr. The red and blue curves in Figure 3d are used to denote the phase reversal of magnetic perturbations along the magnetometer chain at 1545 UT.

Another case of ionospheric equivalent currents associated with penetration electric fields on 17 April 2002 has been reported by Huang (2019a), and a detailed analysis can be found in that paper. Here we only include some measurements of the ionospheric vertical plasma drifts, magnetic fields, and currents of that case for comparison, as shown in Figure 3. The solar wind dynamic pressure is stable, and the IMF *B*_*z*_ has multiple southward turnings between 1100 and 1900 UT, with a quasi period of ~1.5 hr. The equatorial vertical ion drift, measured by the Jicamarca radar, is increased when the IMF turns southward and decreased when the IMF turns northward, which is the typical feature of penetration and shielding electric fields. The yellow and blue bars in Figures 3a–3d denote the peak at 1545 UT and the valley at 1615 UT in the vertical ion drift, and the peak and valley are related to the occurrence of southward IMF (penetration electric field) and northward IMF (shielding electric field), respectively.

Near the geomagnetic dip equator, a zonal electric field in the dayside *E* layer generates vertical Hall currents. Accumulation of charged particles in the upper and low boundaries of the *E* layer causes a vertical polarization electric field, generating a horizontal Hall current which adds to the Pedersen current. Such enhancement in the Pedersen current or conductivity is called the Cowling effect, and this is the mechanism for the generation of strong equatorial electrojet over the magnetic equator on the dayside (e.g., see Kelley, 1989). The electrojet current is typically much stronger than the currents at low and middle latitudes, and the magnetic deviations caused by equatorial electrojet are much larger than those at higher latitudes. In the present case, magnetic field data measured by the latitudinal magnetometer chain in the American sector, depicted in Figure 3d, show multiple peaks caused by the southward turnings of the IMF. The obvious correlation between the IMF southward turnings and the very large increases in the daytime northward magnetic field at HUA, near the magnetic equator, indicates that the eastward electrojet current is enhanced through the Cowling effect. This is another evidence of the occurrence of eastward penetration electric fields in the dayside equatorial region.

Figure 3e shows the equivalent current pattern in the Northern Hemisphere at 1545 UT when the IMF turns southward. The current pattern is characterized by a large counterclockwise vortex on the dayside and evening sector and a clockwise current vortex near dawn. The current pattern at 1615 UT during northward IMF, presented in Figure 3f, is characterized by a large clockwise vortex on the dayside and evening sector and a counterclockwise vortex near dawn. The small current vortex near dawn, the clockwise vortex during southward IMF in Figure 3e and the counterclockwise vortex during northward IMF in Figure 3f, appears to extend into low latitudes in the dawn sector, although the low‐latitude part of the current vortex cannot be clearly identified because of lack of measurements.

**Figure 4:**
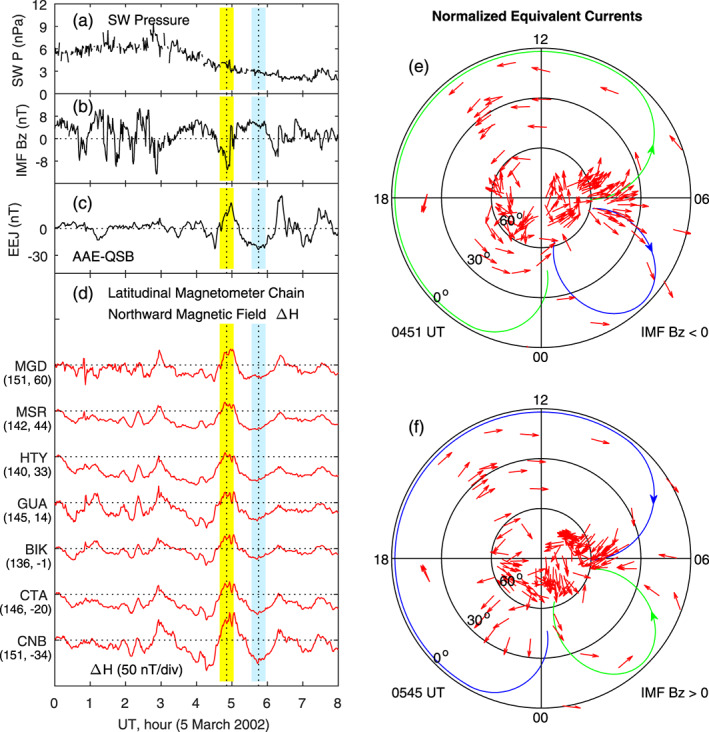
Geomagnetic variations and ionospheric equivalent currents associated with penetration and shielding electric fields caused by southward and northward turnings of the IMF on 5 March 2002. Shown in Figure 4 are (a) solar wind pressure, (b) IMF Bz, (c) equatorial electrojet, (d) magnetic field northward component along the latitudinal magnetometer chain, (e) equivalent currents in the northern hemisphere at 0451 UT for southward IMF, and (f) equivalent currents in the northern hemisphere at 0545 UT for northward IMF, respectively. The vertical yellow and blue bars denote the maximum and minimum in the magnetic field along the latitudinal magnetometer chain in the Asian‐Australian sector. LT along this magnetometer chain is about UT + 10 hr. The longitude and latitude of each magnetometer station are given in the parenthesis underneath the station name.

In the cases of Figures 2 and 3, measurements of vertical ion drift by the Jicamarca radar are used to justify the occurrence of penetration electric fields near noon (around 1700 UT). At this UT, the Pacific Ocean is in the dawn sector, and almost no ground magnetometer data can be used to derive the ionospheric currents at low latitudes. We examined the case on 5 March 2002 when magnetometer data are available at low latitudes in the dawn sector, and the results are presented in Figure 4. The yellow and blue shadings denote an IMF southward turning at 0451 UT and an IMF northward turning at 0545 UT, respectively. The corresponding increase and decrease in the equatorial electrojet (EEJ) in Figure 4c, as well as in the northward magnetic field measured along the latitudinal magnetometer chain in the Asian‐Australian sector on the dayside in Figure 4d, can be viewed as the signature of eastward and westward electric fields. No radar measurements of equatorial ion drifts are available in this case. The large current vortex on the dayside and evening sector in Figures 4e and 4f is similar to that in Figures 2 and 3. The relatively small current vortex in the midnight‐dawn sector, clockwise during southward IMF and counterclockwise during northward IMF, can be clearly seen, providing evidence of a double vortex system of the currents.

Based on the observations of Figures 2, 3, 4, it is concluded that the ionospheric equivalent current system caused by southward IMF, corresponding to the occurrence of penetration electric fields, consists of a large counterclockwise vortex on the dayside and evening sector and a smaller clockwise vortex in the midnight‐dawn sector. During northward IMF, the current pattern is similar, but the polarity of the current vortices is reversed. That is, the equivalent current system caused by northward IMF turning, corresponding to the occurrence of shielding electric fields, consists of a large clockwise vortex on the dayside and evening sector and a smaller counterclockwise vortex in the midnight‐dawn sector.

### 2.2 Current System Caused by Magnetospheric Substorms

Magnetospheric substorms can occur during geomagnetically quiet times and during magnetic storms. In this study, we analyze a specific type of substorms that occur during magnetic storms. The variations of proton flux measured by satellites at geosynchronous orbit are similar to a sawtooth shape, so this type of substorms is often termed sawtooth events or sawtooth oscillations (e.g., Huang, 2002; Huang, Foster, et al., 2003; Huang, Reeves, et al., 2003, 2005). The solar wind pressure and IMF can be very stable across the onset of the substorm, so potential effects caused by sudden changes in the solar wind pressure and IMF should be very small and ignorable. We present the observations of ionospheric currents during two sawtooth events. Direct measurements of ionospheric plasma drifts are not available in these two cases. Penetration electric fields caused by sawtooth substorms have been reported by Huang (2009, 2012) and in other cases by Yamazaki and Kosch (2015), Tulasi Ram et al. (2016), and Hui et al. (2017).

**Figure 5:**
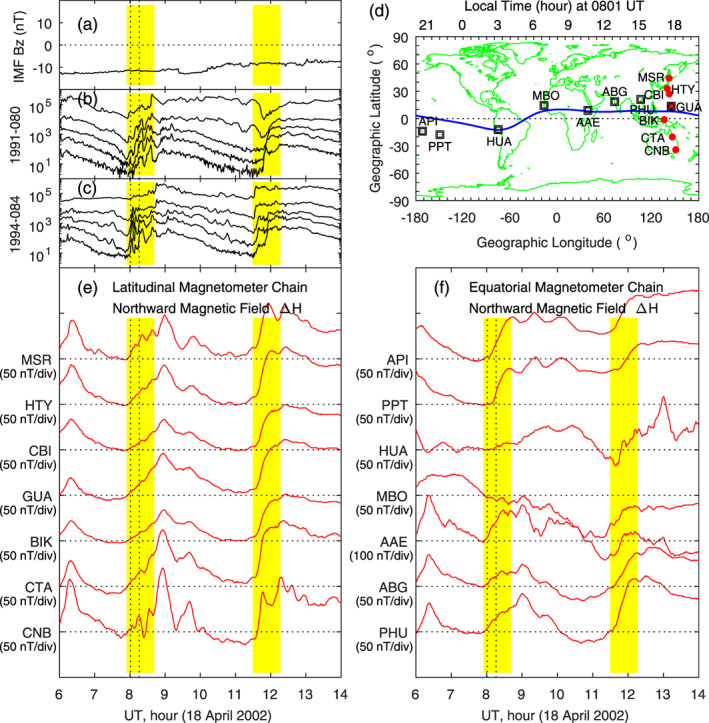
Geomagnetic variations caused by the onset of magnetospheric substorms during a sawtooth event on 18 April 2002. The yellow shading denotes the substorm expansion phase. Shown in Figure 5 are (a) IMF Bz, (b) proton flux measured by the LANL 1991‐080 satellite, (c) proton flux measured by the LANL 1994‐084 satellite, (d) magnetometer stations along the latitudinal chain and along the equatorial chain, (e) magnetic field northward component along the latitudinal magnetometer chain, and (f) magnetic field northward component along the equatorial magnetometer chain, respectively. The vertical dotted lines denote the times at 5 and 20 min after the onset.

The first case is the sawtooth event on 18 April 2002, and this case has been analyzed extensively (Huang, 2002, 2011; Huang & Cai, 2009; Huang, Foster, et al., 2003, 2004, Huang, Reeves, et al., 2005). In this case, the IMF remained continuously southward and stable for more than 24 hr, and the solar wind pressure was also very stable. Multiple space‐based and ground‐based instrumental measurements of magnetospheric and ionospheric parameters are available, and about 10 cycles of quasiperiodic sawtooth substorms are identified. Those studies reveal that the excitation of substorm onset under continuous southward IMF is controlled by the total energy stored in the magnetotail and that an external trigger in the solar wind for substorm onset is not necessary. In the present study, we only show two cycles of the substorm activity on this day. The IMF, plotted in Figure 5a, is southward. Plotted in Figures 5b and 5c are energetic proton flux in energy channels of 50–75, 75–113, 113–170, 170–250, and 250–400 keV, measured by two satellites, LANL 1991‐080 and LANL 1994‐084, at geosynchronous orbit. At 0800 UT, the LANL1991‐080 satellite was at ~2100 LT, and the LANL 1994‐084 satellite was at ~1800 LT. Proton fluxes are injected from the magnetotail to the inner magnetosphere in the evening sector and drift westward. The sudden simultaneous flux increases in all energy channels represent dispersionless injection. The proton flux injection coincides well with the depolarization of the magnetic field in the inner magnetosphere and is often used as the signature of substorm onset. The expansion phase of substorms is denoted by the yellow shading. The first and second onsets occurs at 0756 and 1131 UT, respectively.

We focus on the variations of global magnetic fields during the expansion phase of the first substorm. Two magnetometer chains are marked in Figure 5d. The latitudinal chain in the Asian‐Australian sector is located around 1800 LT at the first onset, and the longitudinal magnetometer chain near the equator covers different time sectors. The northward magnetic fields measured by the latitudinal magnetometer chain, depicted in Figure 5e, and measured by the longitudinal chain, depicted in Figure 5f, all show an increase following the onset. The simultaneous increases of the northward magnetic fields at different latitudes and longitudes/local times, except for HUA and MBO in the postmidnight and dawn sector, indicate occurrence of eastward currents at those locations. The substorm current wedge exists near midnight and causes large auroral electrojet. However, at low latitudes, ionospheric electric fields caused by substorm onset/expansion phase have the same characteristics as penetration electric fields caused by IMF southward turning, eastward on the dayside and westward on the nightside (Huang, 2009, 2012). In the dayside *E* region, the Cowling effect causes enhancement in equatorial electrojet. AAE (−0.06° MLat) is located near local noon during the time of interest, and the magnetic deviation at AAE is larger than those at other local times. The larger amplitude of magnetic field increase at AAE is an indirect evidence of the occurrence of an eastward penetration electric field caused by substorm onset, consistent with previous studies (Huang, 2009, 2012; Yamazaki & Kosch, 2015).

In Figure 5e, the magnetic field at 0756 UT, the time of the substorm onset, is taken to be the reference for each station. That is, the northward magnetic field at 0756 UT is set to be zero for each station. The increase (or decrease) of the magnetic field after the onset is caused by the onset because there is no obvious change in the solar wind pressure and IMF. The two vertical dotted lines denote 0801 and 0816 UT, 5 and 20 min after the onset. The values of the magnetic field at 0801 and 0816 UT are net changes relative to the reference (zero) at 0756 UT.

**Figure 6:**
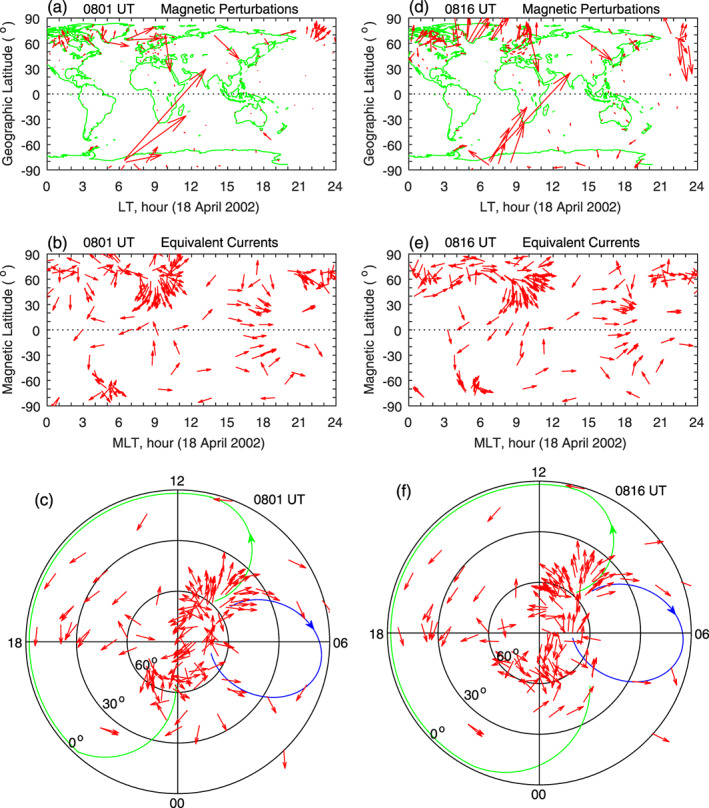
Global geomagnetic variations and ionospheric equivalent currents at 5 and 20 min after the onset of magnetospheric substorm during a sawtooth event on 18 April 2002. Figures 6a–6c show global magnetic perturbations, global equivalent currents, and equivalent currents in the northern hemisphere at 0801 UT, respectively. Figures 6d–6f: Same as Figures 6a–6c but at 0816 UT.

The net changes in the global magnetic fields at 0801 UT are shown in Figure 6a. The magnetic field changes are large at high latitudes, indicating the source locations of the ionospheric currents. The ionospheric equivalent currents derived from the magnetic field changes are plotted in Figure 6b, and the equivalent current pattern is shown in polar coordinates in Figure 6c. It is clear that the current system is characterized by a large counterclockwise vortex covering the dayside and evening sector and a small clockwise vortex near dawn. The right column of Figure 6 shows the magnetic field changes and equivalent currents at 0816 UT, and their distributions are almost the same as those in the left column, indicating that the new current system is very stable. In fact, the stable current system exists for more than 30 min.

**Figure 7:**
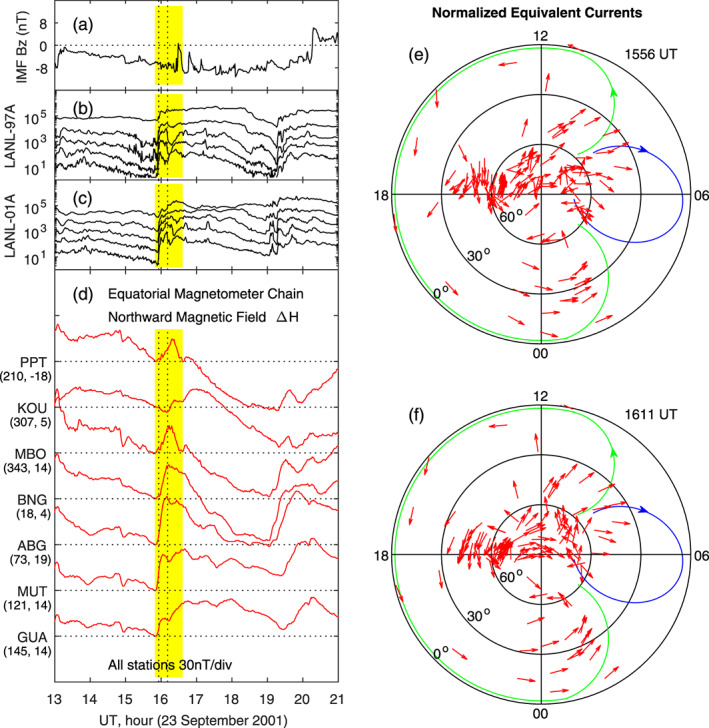
Geomagnetic variations and ionospheric equivalent currents caused by the onset of magnetospheric substorm during a sawtooth event on 23 September 2001. Shown in Figure 7 are (a) IMF Bz, (b) proton flux measured by the LANL‐97A satellite, (c) proton flux measured by the LANL‐01A satellite, (d) magnetic field northward component along the equatorial magnetometer chain, and (e)‐(f) equivalent currents in the northern hemisphere at 1556 and 1611 UT, respectively. The longitude and latitude of each magnetometer station are given in the parenthesis underneath the station name.

Another substorm case on 23 September 2001 is shown in Figure 7. The onset, indicated by the sudden increases in the proton flux measured by the LANL‐97A and LANL‐01A geosynchronous satellites in Figures 7b and 7c, occurs at 1551 UT. The IMF remains southward across the onset, and the solar wind pressure is also stable (not shown here). Plotted in Figure 7d are the northward magnetic fields measured by the longitudinal magnetometer chain near the equator. The magnetic field at the onset (1551 UT) is taken to be the reference (zero) for each station. A sudden increase in the magnetic field following the onset is obvious at all stations. The two vertical dotted lines denote 1556 and 1611 UT. The equivalent current system derived from the net changes of the magnetic field caused by the onset is plotted in Figures 7e and 7f. The current system is characterized by a large counterclockwise vortex on the dayside and evening sector and a small clockwise vortex near dawn, same as the case in Figure 6.

### 2.3 Current System Caused by Sudden Changes in the Solar Wind Pressure

**Figure 8:**
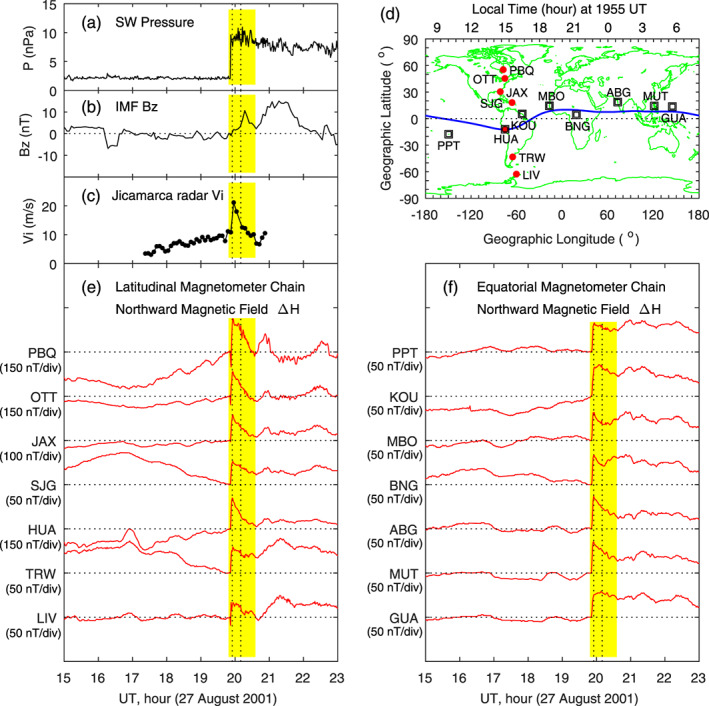
Geomagnetic variations caused by a sudden enhancement in the solar wind dynamic pressure on 27 August 2001. The yellow shading denotes the increase in the magnetic field. Shown in Figure 8 are (a) solar wind pressure, (b) IMF Bz, (c) equatorial vertical ion drifts measured by the Jicamarca radar, (d) magnetometer stations along the latitudinal chain and along the equatorial chain, (e) magnetic field northward component along the latitudinal magnetometer chain, and (f) magnetic field northward component along the equatorial magnetometer chain, respectively. The vertical dotted lines denote the times at 5 and 20 min since the beginning of the sudden increase of the geomagnetic field.

A sudden change, an increase or a decrease, in the solar wind pressure causes a corresponding change in the geomagnetic field. Figure 8 shows the case on 27 August 2001. The solar wind pressure is increased by ~8 nPa within 1 min. The IMF *B*_*z*_ is positive and gradually increases across the discontinuity of the solar wind pressure. A sudden increase in the vertical ion drift is measured by the Jicamarca radar in the dayside equatorial region (~1500 LT), plotted in Figure 8c, and the occurrence of the dayside enhanced vertical ion drift (eastward penetration electric field) is caused by the solar wind pressure enhancement. A detailed analysis of penetration electric fields caused by solar wind pressure enhancements in this case and other similar cases has been made by Huang et al. (2008).

Magnetic field data measured by two magnetometer chains, one along latitude and another along the equator, are plotted in Figures 8e and 8f. The northward magnetic field shows a sudden increase at 1953 UT at all latitudes and local times. We take the magnetic field at 1952 UT, just before the sudden increase, as a reference for each station. That is, the magnetic field is taken to be zero at 1952 UT. Two vertical dotted lines denote 1957 and 2012 UT, 5 and 20 min since the sudden increase in the geomagnetic field. The increase of the magnetic field at 1957 and 2012 UT is the consequence of the solar wind pressure enhancement. It can be seen in Figure 8e that the increase of the magnetic field is large at higher latitudes (PBQ and OTT), indicating the high‐latitude source region of the disturbance currents, and at the magnetic equator (HUA), indicating the Cowling effect in the dayside equatorial ionosphere. Figure 8f shows that the magnetic field caused by the solar wind pressure enhancement has comparable amplitude at all local times.

**Figure 9:**
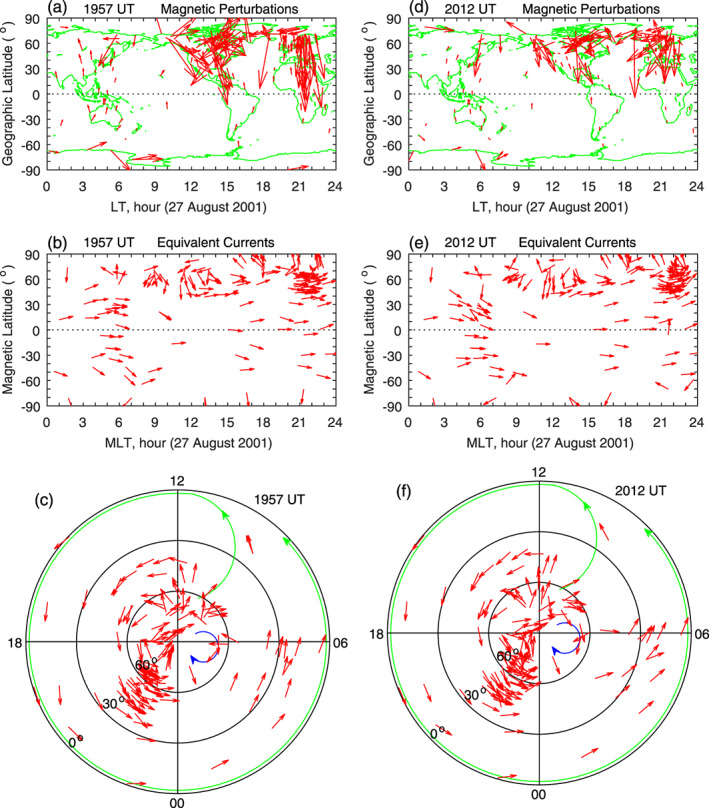
Global geomagnetic variations and ionospheric equivalent currents at 5 and 20 min since the beginning of the sudden increase of the geomagnetic field on 27 August 2001. Figures 9a–9c show global magnetic perturbations, global equivalent currents, and equivalent currents in the northern hemisphere at 1957 UT, respectively. Figures 9d–9f: Same as Figures 9a–9c but at 2012 UT.

Figure 9a shows the global distribution of the magnetic field increases at 1957 UT. Obviously, large magnetic field increases occur at high latitudes. The normalized equivalent currents are plotted in Figure 9b. An interesting feature is that the currents at low latitudes in both the Northern and Southern Hemispheres are all eastward at all local times. The equivalent current system in polar coordinates, plotted in Figure 9c, is characterized by a large, single counterclockwise vortex at middle and low latitudes. In the dawn sector, it appears that there is a very small clockwise current vortex above ~60° MLat, and this clockwise vortex is denoted by the blue curve with arrow. The right column of Figure 9 shows the magnetic field changes and equivalent currents at 2012 UT. Large increases of the magnetic field occur at high latitudes, and the current system consists of a large counterclockwise vortex at middle and low latitudes and a small clockwise vortex above ~60° MLat near dawn, nearly the same as those at 1957 UT.

**Figure 10:**
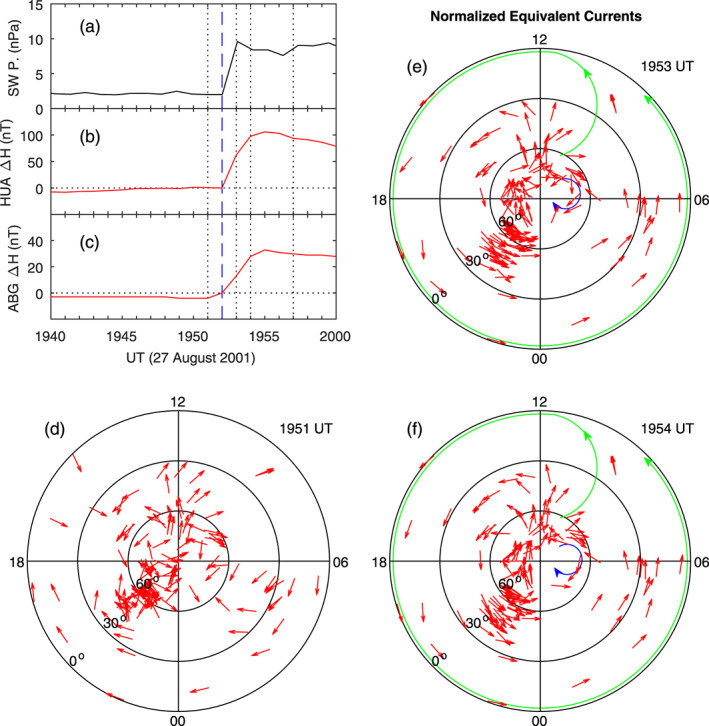
Global ionospheric equivalent currents at 1 min before, and 1 and 2 min after the beginning of the effect of the solar wind pressure enhancement on the geomagnetic field on 27 August 2001. Shown in Figure 10 are (a) solar wind pressure, (b) magnetic field northward component at HUA, (c) magnetic field northward component at ABG, and (d)‐(f) equivalent currents in the northern hemisphere at 1951, 1953, and 1954 UT, respectively.

Because the changes in the solar wind pressure and the subsequent geomagnetic field response occur very quickly, this case provides a very good opportunity to examine how long the ionospheric current system takes to reconstruct. The details are shown in Figure 10. Magnetic field data from two magnetometers are used as example. At 1952 UT, HUA near the magnetic equator is located at ~1600 LT, and ABG at ~12° MLat, is located at ~0100 LT. The magnetic field on both the dayside (HUA) and the nightside (ABG) shows a sudden increase at 1953 UT. The magnetic field at ABG appears to have a small increase between 1951 and 1952 UT; it is not certain whether this small increase is related to the solar wind pressure enhancement.

The equivalent current patterns at 1951, 1953, and 1954 UT are plotted in Figures 10d–10f, respectively. Note that the magnetic field at 1952 UT is taken to be the reference (zero), so all magnetic field changes and currents at 1952 UT are zero. The equivalent currents below 60° MLat at 1951 UT are mostly westward on the nightside and irregular on the dayside. In contrast, the currents at middle and low latitudes are all eastward at 1953 and 1954 UT, and the current system consists of a large counterclockwise vortex below ~60° MLat and a very small clockwise vortex above ~60° MLat near dawn. This current pattern is the same as that at later times (1957 and 2012 UT, shown in Figures 9c and 9f).

**Figure 11:**
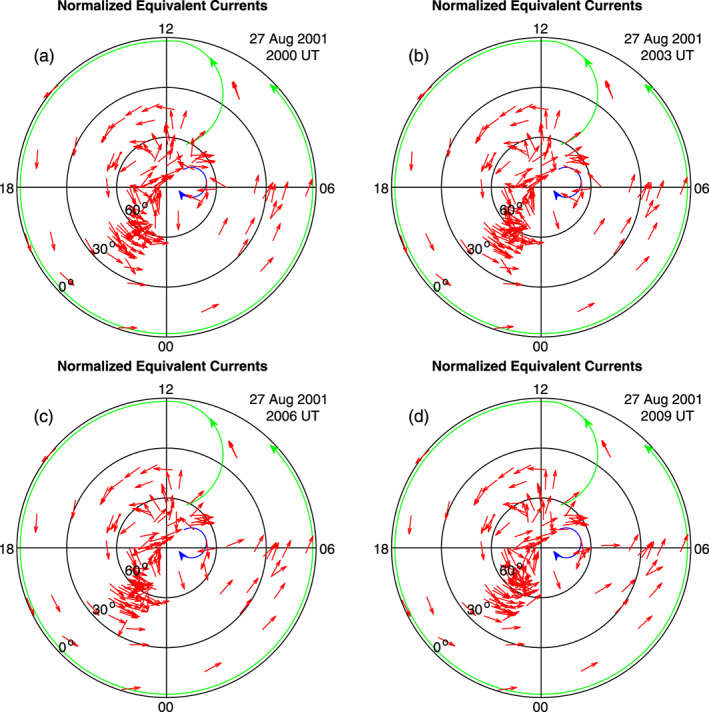
Global ionospheric equivalent currents plotted at every 3 min to show the stability of the current system in the case of 27 August 2001. Figures 11a–11d show equivalent currents in the northern hemisphere at 2000, 2003, 2006, 2009 UT, respectively.

In order to show that the current system remains stable between 1957 and 2012 UT, the current patterns at 2000, 2003, 2006, and 2009 UT are presented in Figure 11. It is obvious that the current patterns in Figure 11 are nearly the same as those in Figures 9 and 10. In the three figures (Figures 9, 10, 11), the current patterns between 1953 and 2012 UT on 27 August 2001 are plotted every 3 min and do not show obvious differences. The results indicate that the current system caused by a sudden increase in the solar wind pressure is established with 1 min, and this new current system is very stable for tens of minutes.

**Figure 12:**
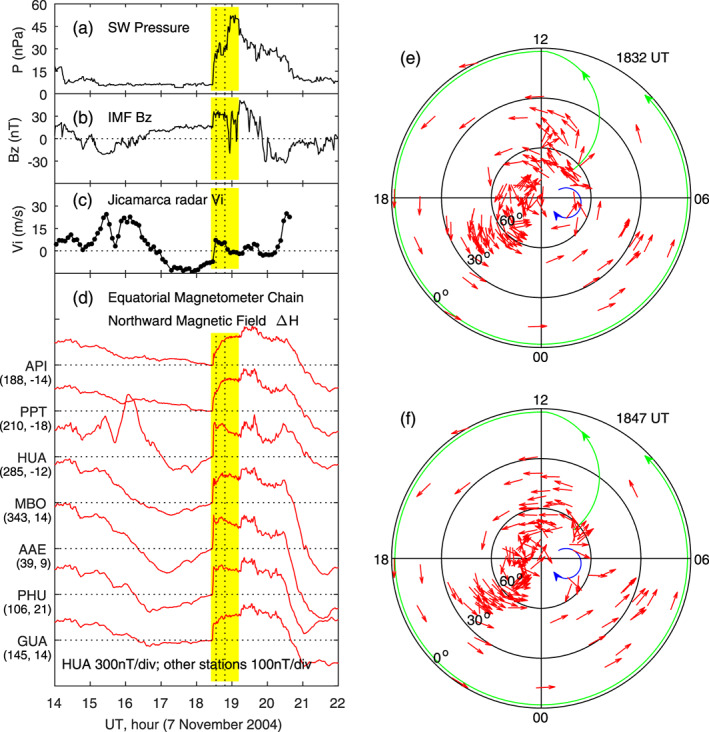
Geomagnetic variations caused by a sudden increase in the solar wind dynamic pressure on 7 November 2004. Shown in Figure 12 are (a) solar wind pressure, (b) IMF Bz, (c) equatorial vertical ion drifts measured by the Jicamarca radar, (d) magnetic field northward component along the equatorial magnetometer chain, and (e)‐(f) equivalent currents in the northern hemisphere at 1832 and 1847 UT, respectively. The vertical dotted lines denote the times at 5 and 20 min since the beginning of the sudden increase of the magnetic field, and the current patterns at these times are given in the right column. The longitude and latitude of each magnetometer station are given in the parenthesis underneath the station name.

An additional example of ionospheric currents in response to a sudden increases of the solar wind pressure is analyzed, and very similar results are found and presented in Figure 12. In the shifted solar wind pressure data, a sudden increase occurs at 1828 UT (Figure 12a); the IMF *B*_*z*_ is positive and enhanced at this moment (Figure 12b). A sudden increase in the vertical plasma drift is detected by the Jicamarca radar, indicating that the solar wind pressure enhancement causes an eastward penetration electric field in the dayside equatorial ionosphere, as shown in Figure 12c. The northward magnetic field shows a sudden increase at all longitudes in response to the solar wind pressure impulse, as measured by the equatorial magnetometer chain (Figure 12d). The ionospheric current patterns at 1832 and 1847 UT, 5 and 20 min after the solar wind pressure increase, are given in Figures 12e and 12f. Obviously, the current system in this case is very similar to the one in the other case (Figures 9, 10, 11) and characterized by a large counterclockwise vortex at middle and low latitudes and a very small clockwise vortex at high latitudes near dawn.

**Figure 13:**
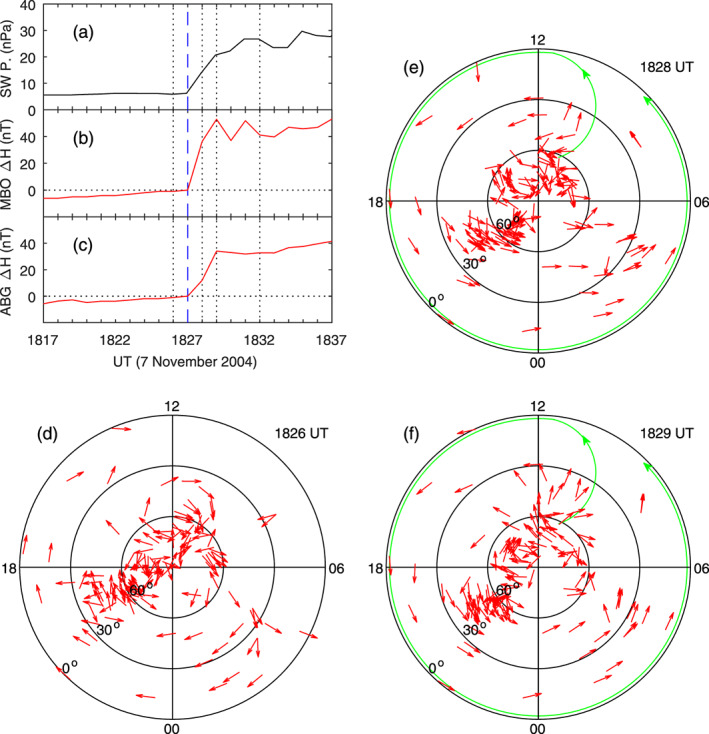
Global ionospheric equivalent currents at 1 min before, and 1 and 2 min after the beginning of the effect of the solar wind pressure enhancement on the geomagnetic field on 7 November 2004, respectively. Shown in Figure 13 are (a) solar wind pressure, (b) magnetic field northward component at MBO, (c) magnetic field northward component at ABG, and (e)‐(f) equivalent currents in the northern hemisphere at 1826, 1828, and 1829 UT, respectively.

This case can also be used to examine the time scale of ionospheric current system reconstruction. In Figure 13, the detailed variations of the magnetic field and currents across the sudden changes are given. The magnetic field at MBO and ABG, which are located in the afternoon and midnight sectors during the period of interest, respectively, changes gradually before 1827 UT and shows a sudden increase after 1827 UT. In the calculation of current changes, the magnetic field at 1827 UT is taken to be the reference (zero), so the ionospheric currents are zero at this moment. The current pattern is largely a clockwise vortex at 1826 UT but changes to a well‐defined counterclockwise vortex at middle and low latitudes at 1828 and 1829 UT. The potential small clockwise current vortex at high latitudes near dawn is not obvious in this case, so it is not denoted. The change of the current system from zero at 1827 UT to the counterclockwise vortex at 1828 UT occurs within 1 min. The current distributions before the reference time and after the reference time in this case are very similar to those in the case of Figure 10, providing further justification that the ionospheric current reconstruction occurs within 1 min.

**Figure 14:**
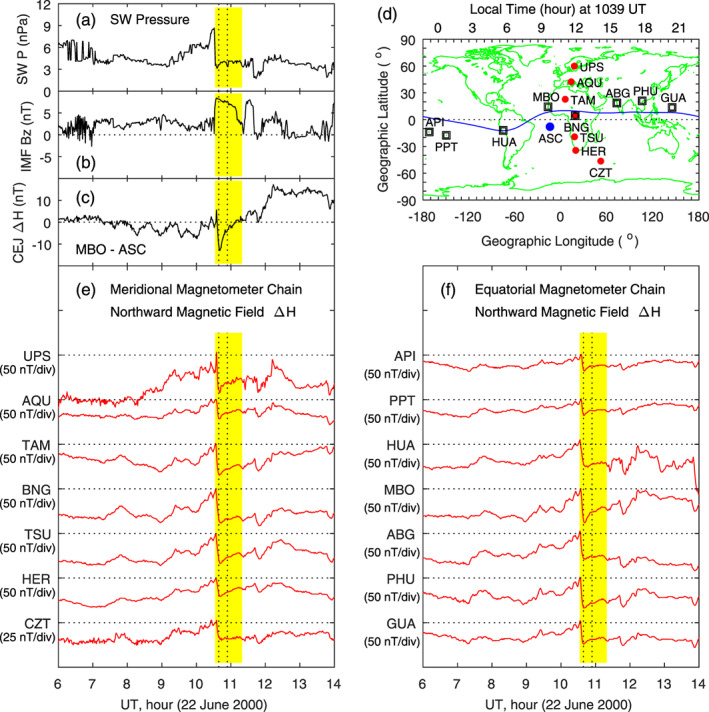
Geomagnetic variations caused by a sudden decrease in the solar wind dynamic pressure on 22 June 2000. The yellow shading denotes the decrease in the magnetic field. Shown in Figure 14 are (a) solar wind pressure, (b) IMF Bz, (c) equatorial counter‐electrojet, (d) magnetometer stations along the latitudinal chain and along the equatorial chain, (e) magnetic field northward component along the latitudinal magnetometer chain, and (f) magnetic field northward component along the equatorial magnetometer chain, respectively. The vertical dotted lines denote the times at 5 and 20 min since the beginning of the sudden decrease of the magnetic field.

A sudden decrease in the solar wind pressure also causes large changes in the geomagnetic field and ionospheric currents, and an example is presented in Figure 14. In this case, the solar wind pressure is suddenly decreased by ~4 nPa at 1034 UT. The IMF *B*_*z*_ is positive prior to the solar wind pressure drop and becomes more positive after the solar wind pressure drop. As can be seen in Figure 14b, a second increase of the IMF *B*_*z*_ occurs at the right edge of the yellow‐shaded interval. This second *B*_*z*_ increase is similar to the first *B*_*z*_ increase but does not have a simultaneous solar wind pressure drop. As shown in Figures 14e and 14f, a large decrease in the northward magnetic field occurs only when there is a solar wind pressure drop. It is therefore reasonable to assume that the sudden decreases in the geomagnetic field, as well as in ionospheric currents analyzed below, are caused by the solar wind pressure drop, rather than by the increase in the northward IMF. The magnetic fields at all latitudes and longitudes/local times show a sudden decrease at 1035 UT. Similar to the case of solar wind pressure increase, the magnetic field at 1034 UT, just before the sudden decrease, is taken to be the reference (zero).

In this case, no measurements of equatorial ionospheric ion drifts/electric fields are available. As shown in the cases of Figures 8 and 12, as well as in other cases reported by Huang et al. (2008), a sudden increase in the solar wind pressure causes an eastward penetration electric field in the dayside equatorial ionosphere. If we assume that an opposite change in the solar wind pressure causes an opposite change in the ionosphere, a sudden decrease in the solar wind pressure is expected to cause a westward penetration electric field in the dayside equatorial ionosphere. Counter electrojet is often used as an indication of westward electric fields (e.g., Kikuchi et al., 2003; Yamazaki & Kosch, 2015). In this case, a magnetometer pair in the morning sector, Mbour (MBO, 343.03°E, 14.38^o^ N, 1.2° MLat) and Ascension Island (ASC, 345.62°E, 7.95°S, −16.34° MLat), are used to calculate counter electrojet. Figure 14c shows the difference in the northward magnetic field between MBO and ASC, and this difference Δ*H* represents electrojet or counter electrojet. The negative Δ*H* following the sudden drop in the solar wind pressure indicates the occurrence of counter electrojet (or westward electric field). No magnetometer measurements at the magnetic equator near noon or in the afternoon sector are available for calculating electrojet or counter electrojet in this case.

**Figure 15:**
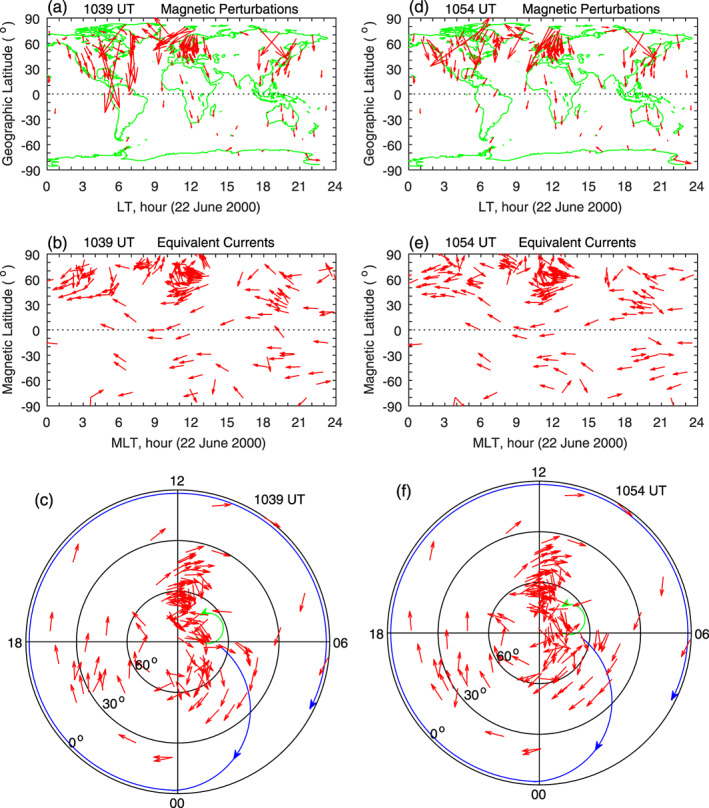
Global geomagnetic variations and ionospheric equivalent currents at 5 and 20 min since the beginning of the sudden decrease of the magnetic field on 22 June 2000. Figures 15a–15c show global magnetic perturbations, global equivalent currents, and equivalent currents in the northern hemisphere at 1039 UT, respectively. Figures 15d–15f: Same as Figures 15a–15c but at 1054 UT.

The two vertical dotted lines in Figure 14 denote 1039 and 1054 UT, 5 and 20 min since the magnetic field starts to decrease. Figure 15 shows the global magnetic field changes (the top row), the equivalent currents in MLT‐MLat coordinates (the middle row), and the equivalent currents in polar coordinates (the bottom row) at these times. It can be seen in the middle panels that the current vectors are all westward at middle and low latitudes. The current pattern in polar coordinates consists of a large clockwise vortex at middle and low latitudes at all local times and possibly a small counterclockwise vortex above ~60° MLat near dawn. Comparing the results in Figures 9 and 15, it is obvious that a sudden increase in the solar wind pressure causes a counterclockwise current vortex at middle and low latitudes and that a sudden decrease in the solar wind pressure causes a clockwise current vortex, verifying that opposite changes in the solar wind pressure cause opposite changes in the ionospheric currents.

**Figure 16:**
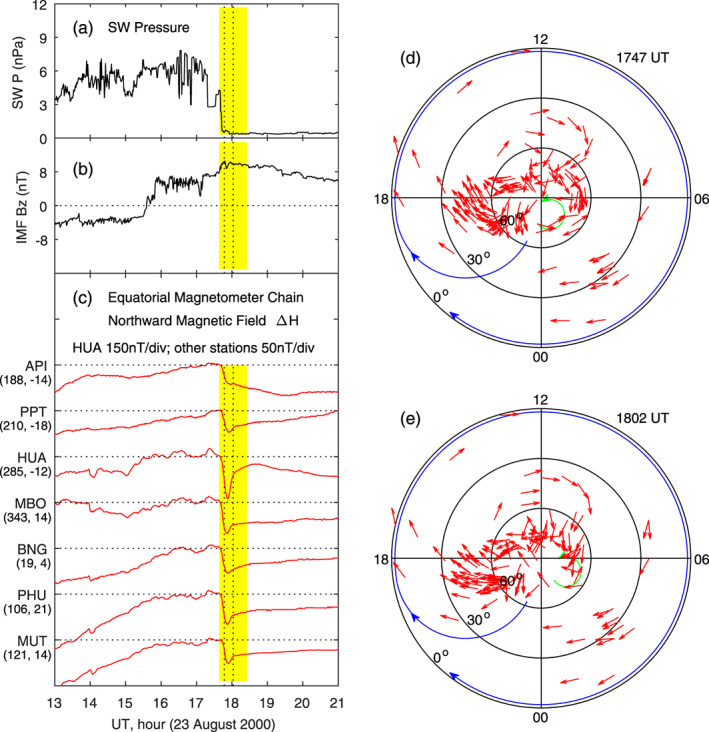
Geomagnetic variations caused by a sudden decrease in the solar wind dynamic pressure on 23 August 2000. Shown in Figure 16 are (a) solar wind pressure, (b) IMF Bz, (c) magnetic field northward component along the equatorial magnetometer chain, and (d)‐(e) equivalent currents in the northern hemisphere at 1747 and 1802 UT, respectively. The vertical dotted lines denote the times at 5 and 20 min since the beginning of the sudden decrease of the magnetic field, and the current patterns at these times are given in the right column.

Another example of the ionospheric current system caused by a sudden decrease in the solar wind pressure is presented in Figure 16. In this case, the solar wind pressure has a sudden decrease while the IMF *B*_*z*_ remains positive and stable. Figure 16c shows that a decrease in the northward magnetic field occurs at all longitudes at low latitudes, measured by the equatorial magnetometer chain. HUA is near local noon during the period of interest. Note that the magnetic field deviation is plotted at 150 nT/div at HUA but 50 nT/div at other stations. The much larger decrease of the northward magnetic field at HUA after the solar wind pressure drop is an indirect evidence of occurrence of an enhanced counter electrojet, as well as a westward electric field, in the dayside equatorial region. Figures 16d and 16e display the current distributions 5 and 20 min after the solar wind pressure drop. The current system is characterized by a large clockwise vortex at middle and low latitudes, very similar to the current system in Figure 15.

### 2.4 Current System Caused by ULF Waves

**Figure 17:**
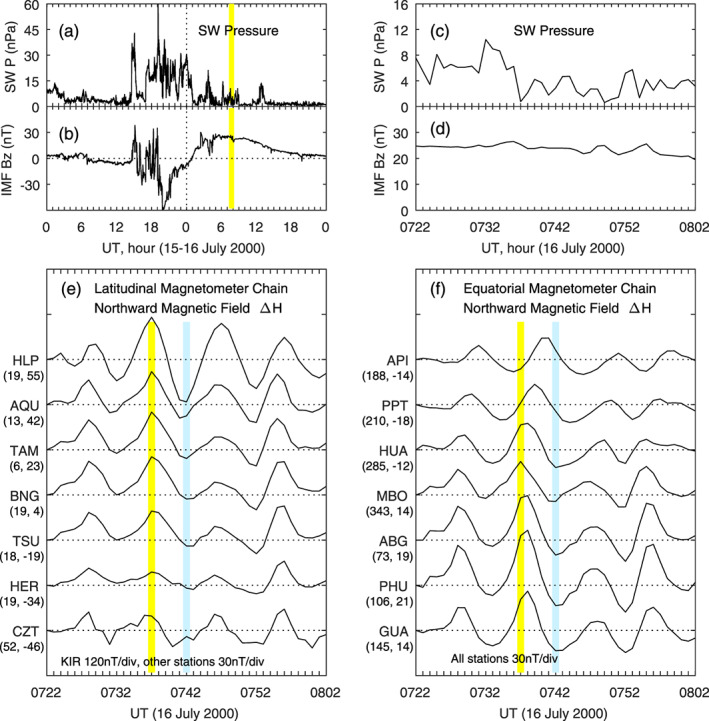
Pc5 pulsations in the geomagnetic field on 16 July 2000. Shown in Figure 17 are (a) solar wind pressure on 15‐16 October 2000, (b) IMF Bz on 15‐16 October 2000, (c) solar wind pressure at 0722‐0802 UT on 16 October 2000, (b) IMF Bz at 0722‐0802 UT on 16 October 2000, (e) magnetic field northward component along the latitudinal magnetometer chain, and (f) magnetic field northward component along the equatorial magnetometer chain, respectively. The vertical yellow and blue bars denote a peak and a valley of the Pc5 pulsations, respectively. The longitude and latitude of each magnetometer station are given in the parenthesis underneath the station name.

Pc5 pulsations, as well as Pc3, Pc4, and Pi2 pulsations, are often observed at low latitudes. For our purpose, we need to find large‐amplitude Pc5 pulsations in which the peak‐to‐valley difference of the magnetic field at middle and low latitudes can be 30 nT or larger, so the difference current distributions at the pulsation peaks and valleys can be unambiguously determined. We searched magnetometer data in 2000–2003 at solar maximum and selected two cases in which the pulsation amplitude is very large. The first Pc5 pulsation case is presented in Figure 17. Figures 17a and 17b show the solar wind pressure and IMF *B*_*z*_ on 15–16 July 2000. The strong southward IMF after ~1900 UT on 15 July causes a severe magnetic storm with a minimum Dst of −301 nT. The Pc5 pulsations of interest occur during the recovery phase of the storm, denoted by the yellow shading in Figures 17a and 17b. Figures 17c and 17d show the details of the solar wind pressure and IMF *B*_*z*_ between 0722 and 0802 UT on 16 July. The IMF *B*_*z*_ during this interval is strongly positive, and the solar wind pressure has some irregular variations but not periodic wave activities.

Figure 17e shows the northward magnetic field in the Pc5 pulsation event measured by the latitudinal magnetometer chain in the African sector (the same chain as in Figure 14d with HLP replacing UPS); this latitudinal chain is in the morning sector during 0722–0802 UT. A moving window of 30 min is used to remove the mean value of the magnetic field, and plotted in Figures 17e and 17f are the magnetic field perturbations induced by the Pc5 pulsations. The moving window of 30 min covers about three cycles of the waves, the mean value over the moving window should fairly represent the magnetic field without the waves. Four cycles of the pulsations are obvious, with peaks at 0728, 0737, 0746, and 0755 UT and valleys at 0733, 0742, 0752, and 0759 UT, respectively. There is a clear periodicity of ~9 min. The peaks and valleys along the latitudinal chain occur nearly simultaneously, within the data resolution of 1 min. We select the peak at 0737 and the valley at 0742 as examples, as denoted by the vertical yellow and blue lines in Figure 17e. No plasma drift measurements at low latitudes are available in this case. Signatures of penetration electric fields measured with HF Doppler at low latitudes during a global Pc5 event were reported by Motoba et al. (2004). In a case in which the ionospheric DP 2 currents showed a quasiperiodic oscillation with a period of ~25 min, HP Doppler radar also detected penetration electric fields in the dusk sector (Abdu, Sastri, et al., 1998).

Figure 17f shows the pulsation activity along the equatorial magnetometer chain. These magnetometers essentially cover all longitudes (local times), and the separation between consecutive stations is, on average, 45° in longitude (3 hr in local time). A prominent feature in this case is that large‐amplitude pulsations occur on both the dayside and nightside. For example, during the period of interest, ABG is near local noon, and HUA is in the postmidnight sector. The peak‐to‐valley change between 0737 and 0742 UT is 39.5 nT at ABG and 29.4 nT at HUA, respectively. The pulsation amplitude is larger at ABG near noon and at PHU and GUA in the afternoon sector, possibly caused by higher ionospheric conductivity in this local time range. The peaks and valleys at API and PPT in the evening sector appear to occur later than other longitudes/local times. This apparent shift in the wave peak/valley is caused by the phase distribution of the pulsations along longitude but does not represent a propagation delay. The wave characteristics of global Pc5 pulsations will be analyzed in detail in a separate study.

**Figure 18:**
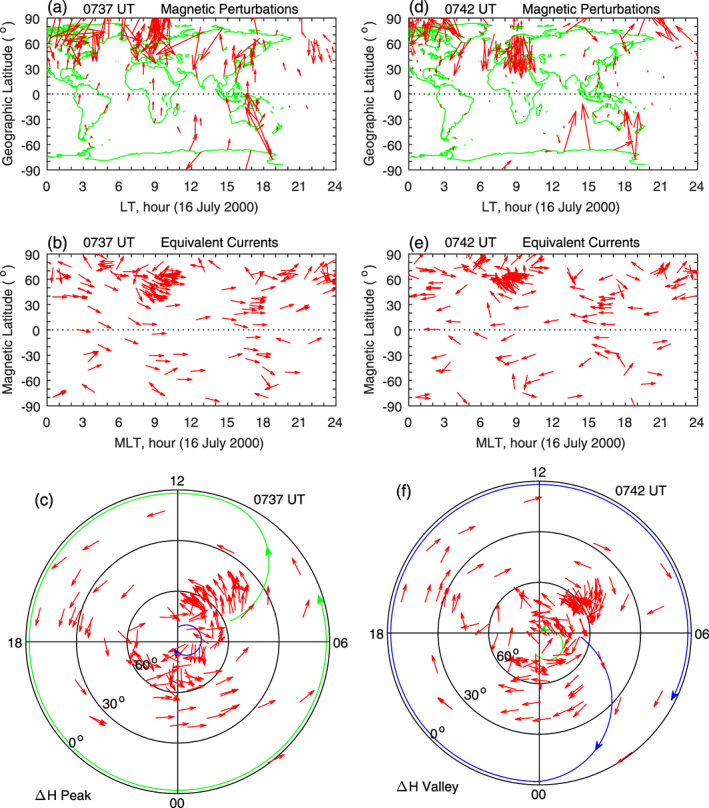
Global geomagnetic variations and ionospheric equivalent currents at the peak and valley of the Pc5 pulsations on 16 July 2000. Figures 18a–18c show global magnetic perturbations, global equivalent currents, and equivalent currents in the northern hemisphere at the 0737 UT pulsation peak, respectively. Figures 18d–18f: Same as Figures 18a‐18c but at the 0742 UT pulsation valley.

Figures 18a–18c in the left column show the global magnetic field perturbations, the equivalent currents in MLT‐MLat, and the equivalent currents in polar coordinates at the 0737 UT peak of the pulsations. Large magnetic perturbations occur at high latitudes, indicating the source of the global magnetic pulsations. The current vectors are all eastward at middle and low latitudes. In polar coordinates, the current pattern in Figure 18c consists of a large, single counterclockwise vortex at middle and low latitudes, covering all local times, and a small clockwise vortex at very high latitudes in the midnight‐dawn sector. The right column of Figure 18 shows the observations at the 0742 UT valley of the pulsations. The source of the global pulsations is still located at high latitudes. Compared with Figure 18c, the current pattern at the pulsation valley is similar to that at the pulsation peak, but the current directions are reversed. The current pattern at the pulsation valley consists of a large clockwise vortex at middle and low latitudes, covering all local times, and a small counterclockwise vortex at high latitudes in the midnight‐dawn sector.

**Figure 19:**
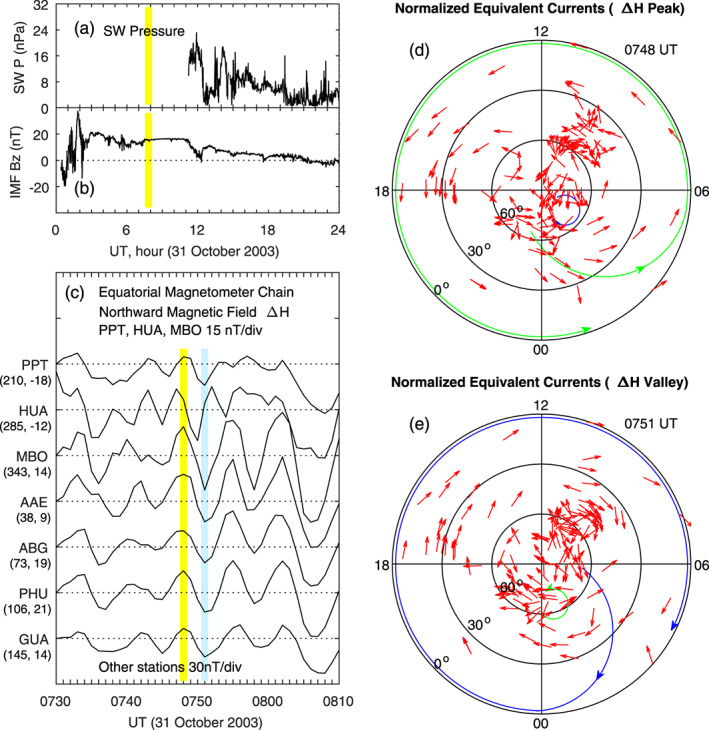
Pc5 pulsation event on 31 October 2003. Shown in Figure 19 are (a) solar wind pressure, (b) IMF Bz, (c) magnetic field northward component along the equatorial magnetometer chain, equivalent currents in the northern hemisphere at the 0748 UT pulsation peak, and (e) equivalent currents in the northern hemisphere at the 0751 UT pulsation valley, respectively. The vertical yellow and blue bars denote a peak and a valley of the Pc5 pulsations, respectively. The longitude and latitude of each magnetometer station are given in the parenthesis underneath the station name. The global ionospheric equivalent currents at the peak and valley of the Pc5 pulsations are shown in the right column.

A second case of large‐amplitude Pc5 pulsations on 31 October 2003 is shown in Figure 19. In this case, solar wind pressure data are not available for the period of pulsations, and the IMF *B*_*z*_ is strongly positive. The magnetic field measurements along the equatorial magnetometer chain is plotted in Figure 19c, and a peak at 0748 UT and a valley at 0751 UT of the pulsations are denoted by the yellow and blue lines, respectively. The normalized equivalent currents in polar coordinates are plotted in Figures 19d and 19e. The current system at the pulsation peak (0748 UT) consists of a large counterclockwise vortex at middle and low latitudes covering all local times and possibly a clockwise vortex at high latitudes near dawn in the midnight‐dawn sector. At the pulsation valley (0751 UT), the current pattern is similar, but the polarity of the current vortices is reversed. The global current distribution and its variation between the peak and valley in this case are very similar to those in the case of Figure 18.

### 2.5 Latitudinal Distribution of Magnetic Field Perturbations

In the previous subsections, magnetic perturbations along some latitudinal and equatorial magnetometer chains are presented. Note that these stations are located in a limited latitudinal range at middle and low latitudes. In addition, different scales are used for different stations to better show the magnetic perturbations caused by external processes. This may cause an impression that the magnetic perturbations at all latitudes have similar amplitude.

**Figure 20:**
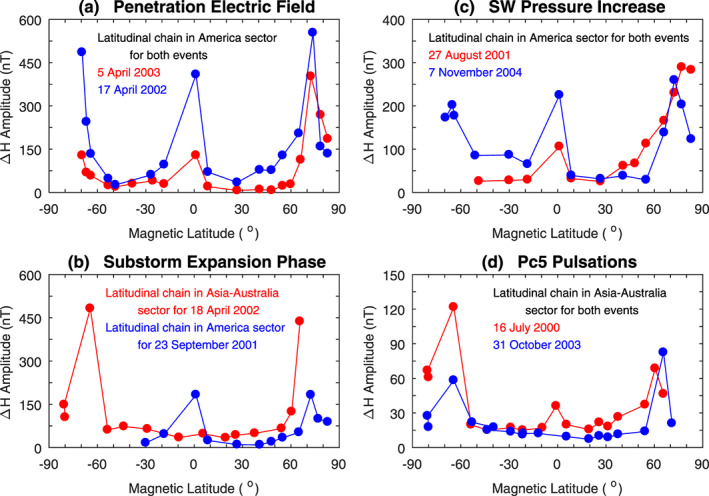
Latitudinal distribution of the amplitude of geomagnetic field perturbations caused by (a) IMF southward turning, (b) substorm onset, (c) sudden change in the solar wind dynamic pressure, and (d) Pc5 pulsations.

In fact, magnetic perturbations in all cases are large at high latitudes, become much smaller at middle latitudes, and are enhanced by the Cowling effect over the magnetic equator. This feature can be seen in the global distribution of unnormalized magnetic perturbations in each case. In order to explicitly show the latitudinal variation of magnetic perturbations, the amplitude of the net magnetic field change in response to each solar or magnetospheric process is plotted as a function of magnetic latitude in Figure 20. The magnetometer chain for each case is denoted in the global map (e.g., Figure 1d). In Figure 20, the magnetometer chain has been extended into the highest latitudes when data are available. All the measurements of the magnetometer chain are made on the dayside or near dusk in these cases. The magnetometer chains in previous figures are plotted in geographic coordinates. Figure 20 is plotted in magnetic latitude to better show the large magnetic deviations in the auroral zones and the enhancement over the magnetic equator.

In the cases of penetration electric fields, the amplitude of the magnetic perturbation at each station is defined to be the net increase from the minimum before the IMF southward turning to the maximum after the IMF southward turning. In the cases of magnetospheric substorms, the amplitude of the magnetic perturbation is the net increase from the substorm onset to the maximum after the onset. In the cases of solar wind pressure changes, the amplitude of the magnetic perturbation is the net increase from the undisturbed reference to the maximum after the solar wind pressure enhancement or the net decrease from the undisturbed reference to the minimum after the solar wind pressure drop. In the case of Pc5 pulsations, the amplitude of the magnetic perturbation is the difference between the peak and valley divided by two. It should be noted that the magnetic perturbations can be in opposite phase across the auroral zone. The absolute value of the net change of the magnetic field is taken to be the amplitude, so the plotted amplitude is always positive.

In Figure 20a, magnetic field data between −60° and 90° MLat are measured by the latitudinal magnetometer chain in the American sector, as denoted in Figure 1d. In the Southern Hemisphere, no magnetometer data are available at high latitudes in the American sector. The data at the three stations between −80° and −60° MLat, the three data points at the left end of Figure 20a, are taken in the Atlantic sector, about 40° east to the American sector. During the period of interest, the latitudinal magnetometer chain in the American sector is near local noon, and the three magnetometers in the Atlantic sector are at about 1500 LT. Large‐amplitude magnetic deviations occur in the auroral zones (between 60° and 80° or between −60° and −80° MLat) in both hemispheres and over the magnetic equator. Similarly, the three data points between −60° and −80° MLat at the left end of Figure 20c in the case of 7 November 2004 are also taken from measurements of three magnetometers in the Atlantic sector. In the case of 23 September 2001 in Figure 20b and in the case of 27 August 2001 in Figure 20c, no measurements are available at high latitudes in the Southern Hemisphere along or near the selected magnetometer chain.

The latitudinal distributions of the magnetic perturbation amplitude are similar in all cases: penetration electric fields (Figure 20a), substorm expansion phase (Figure 20b), sudden increase in the solar wind pressure (Figure 20c), and Pc5 pulsations (Figure 20d). Large peaks occur at the auroral latitudes (60–80° MLat), indicating the source region where the FACs flow into the ionosphere. The enhancement of the magnetic perturbations over the magnetic equator is caused by the Cowling effect. The magnetic perturbations are much smaller at middle and low latitudes, and the variation of the amplitude is also small in this limited latitude range, as presented in the individual cases.

The variation of geomagnetic field perturbations with latitude is investigated in some previous studies. Kikuchi et al. (1996) derived the latitudinal profile of the magnitude of DP 2 fluctuations between 0 and 80° MLat in a case of penetration electric fields. The profile in their case is similar to our result of Figure 20a but has a smaller amplitude than that in our cases, perhaps related to the strength of the driving source. Kikuchi et al. (2000) derived the latitudinal profile of the amplitude of negative bays between 0 and 60° MLat in a substorm case, and the amplitude of the magnetic fluctuations at the auroral or equatorial latitudes in their case is smaller than that in our cases (Figure 20b). The difference could be related to the different substorm types: an isolated substorm in their case but sawtooth substorms during magnetic storms in our cases.

## 3 Illustrative Current Patterns Caused by Different Solar and Magnetospheric Processes

**Figure 21:**
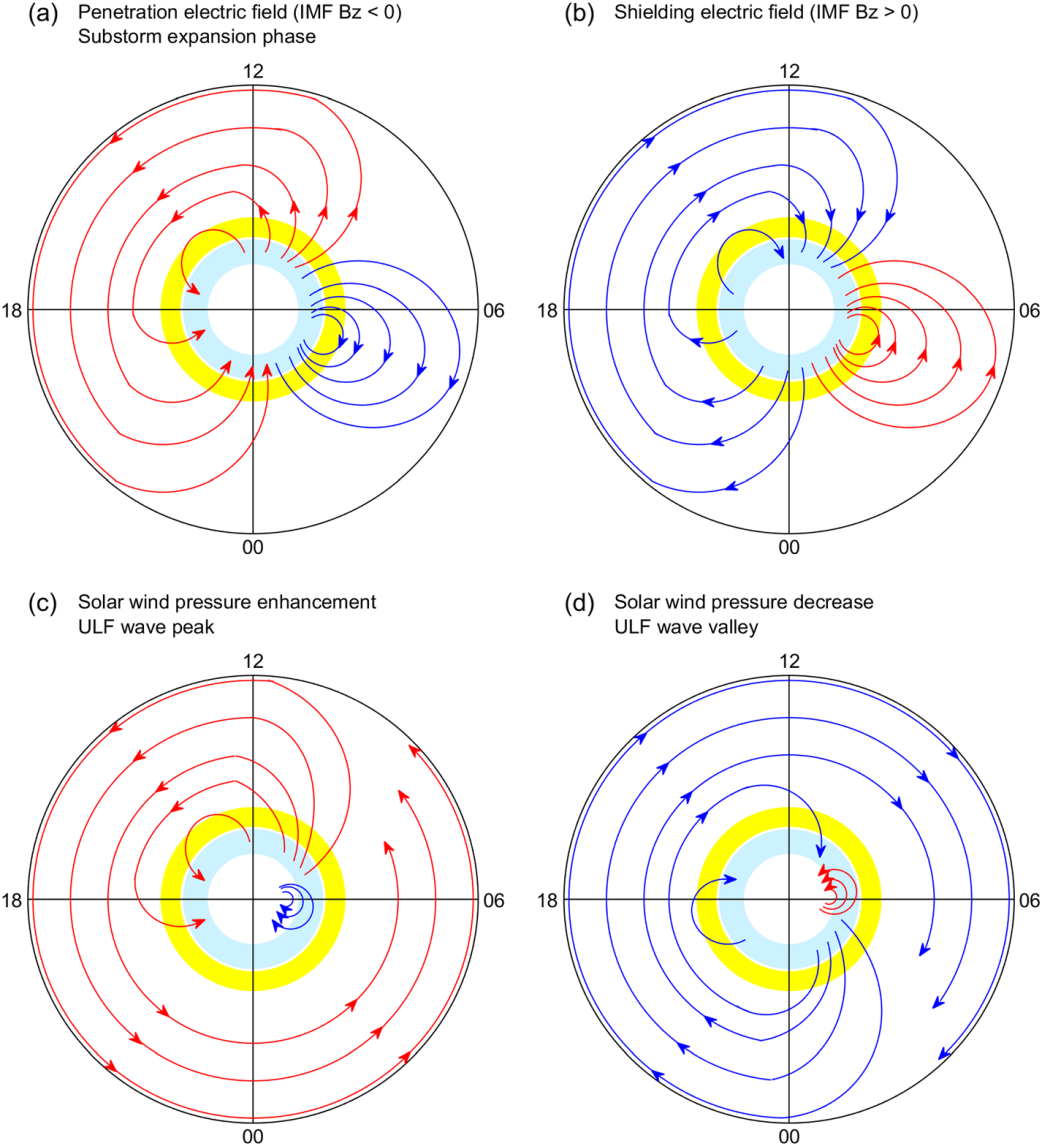
Illustrations of the ionospheric equivalent disturbance current systems caused by IMF reorientations, magnetospheric substorms, solar wind pressure enhancement and decrease, and ULF waves, respectively. This figure is plotted in magnetic local time‐magnetic latitude coordinates, and the black outer circle represents the magnetic equator. Figure 21 shows (a) equivalent current system caused by penetration electric fields and substorm expansion phase, (b) equivalent current system caused by shielding electric fields, (c) equivalent current system caused by solar wind pressure enhancement and ULF wave peak, and (d) equivalent current system caused by solar wind pressure decrease and ULF wave valley, respectively. The blue and yellow rings at high latitudes denote the approximate locations of the Regions 1 and 2 field‐aligned currents at ionospheric heights.

The observed equivalent current patterns driven by different processes are presented in section 2. It is not difficult to find that some current patterns are very similar. Based on the observations, the current patterns can be categorized into four types and are illustrated in Figure 21. In this figure, counterclockwise current vortex is plotted in red, and clockwise current vortex is plotted in blue. Note that the red current vortex in Figure 21 corresponds to the current vortex outlined by the green curve in previous figures. The heavy light blue and yellow rings in Figure 21 denote the approximate locations of the Regions 1 and 2 FACs at ionospheric heights but do not imply that the FACs are uniformly distributed along the rings. More details of the FACs can be found in Iijima and Potemra (1978).

Figure 21a represents the current pattern caused by IMF southward turning (occurrence of penetration electric fields) and by the onset of magnetospheric substorms; the corresponding observations are shown in Figures 2c, 3e, 4e, 6c, 6f, 7e, and 7f. Figure 21b represents the current pattern caused by IMF northward turning (occurrence of shielding electric fields); the corresponding observations are shown in Figures 2f, 3f, and 4f. Figure 21c represents the current pattern caused by a sudden enhancement in the solar wind pressure or by Pc5 pulsations at the pulsation peak; the corresponding observations are shown in Figures 9c, 9f, 10e, 10f, 11a–11d, 12e, 12f, 13e, 13f, 18c, and 19d Figure 21d represents the current pattern caused by a sudden decrease in the solar wind pressure or by Pc5 pulsations at the pulsation valley; the corresponding observations are shown in Figures 15c, 15f, 16d, 16e, 18f, and 19e.

It should be mentioned that the purpose of Figure 21 is to show the overall global pattern and direction of the ionospheric equivalent currents. The relative size and location of the current vortices can be highly variable from case to case. For example, the relatively small clockwise current vortex caused by IMF southward turning (Figures 2c and 3e) and the relatively small counterclockwise current vortex caused by IMF northward turning (Figures 2f and 3f) appear to be near dawn. In contrast, in Figures 4e and 4f, these current vortices are obviously located in the midnight‐dawn sector. The current contours are used to indicate the direction of the currents but do not imply that the current intensity is the same between any two consecutive contours. In the method for deriving the equivalent currents, the current strength is proportional to the amplitude of the magnetic perturbation at any location, and the latitudinal variation of the current strength on the dayside is similar to that for the magnetic perturbations given in Figure 20.

The equivalent current system consists of currents from different sources. We take Pc5 pulsations as example. Pc5 pulsations are excited in the magnetosphere and have periods of 150–600 s. Another type of long‐period pulsations is Pi2 pulsations that have periods of 40–150 s. Pi2 pulsations originate in the magnetosphere, often accompanying the onset of the substorm expansion phase and have been observed in low‐latitude and equatorial regions (e.g., Imajo et al., 2016). A detailed analysis of the sources of ionospheric currents caused by Pi2 pulsations is helpful for understanding the sources of the ionospheric currents caused by Pc5 pulsations. Imajo et al. (2017) used a global magnetospheric‐ionospheric current model to calculate the equivalent currents caused by Pi2 pulsations. In that model, the dayside equivalent currents are primarily from the ionospheric currents and eastward at middle and low latitudes, and the nightside equivalent currents are primarily from the FACs and also eastward at middle and low latitudes (Figure 3 of Imajo et al.). The only exception is the dawn sector where the equivalent currents have significant equatorward component in both hemispheres. In our observations, the equivalent currents caused by Pc5 pulsations show the same features. In Figure 18b, the equivalent currents have equatorward components (pointing southeast in the Northern Hemisphere and northeast in the Southern Hemisphere) between 0300 and 0600 MLT and are mostly eastward at low latitudes at all other local times.

The equatorward/poleward currents near dawn occur not only during ULF waves but also during other processes. This feature is obvious in Figures 3e and 4e after an IMF southward turning (occurrence of penetration electric fields) and in Figures 6c, 6f, 7e, and 7f during the substorm expansion phase. The equatorward currents near dawn imply that the FACs make important contribution to the equivalent currents and the double vortex system (depicted in Figures 21a and 21b) in the cases of penetration electric fields and substorm onset.

Besides the FACs, the ring current may be also important for the case of substorms because energetic protons are injected into the ring current during the substorm expansion phase, and the magnetopause currents become particularly significant when a sudden enhancement in the solar wind pressure impacts on the magnetosphere. The equivalent currents include contributions from all the sources: the ionospheric currents, the FACs, the ring current, and the magnetopause currents. The different sources and the different distribution of the FACs with local time and latitude during different processes cause the current systems illustrated in Figure 21. The primary objective of this paper is to identify the global distribution of the equivalent currents. The relative importance of different current sources in contributing to the equivalent currents and their latitudinal variation is beyond the scope of this study.

The current patterns Figure 21 have some similarities and also some differences. The similarities are a large vortex on the dayside and evening sector and a small vortex near dawn. The differences are the relative size and local time coverage of the two vortices. We use “double vortex current system” and “single vortex current system” to represent the different current distributions. The current pattern in Figures 21a and 21b consists of a large vortex and a relatively small vortex; we term this current system as a double vortex system. The current pattern in Figures 21c and 21d is characterized by a single vortex at middle and low latitudes; we term this as a single vortex system, although a very small vortex exists at high latitudes near dawn.

As already mentioned at the beginning of this paper, the observed current patterns in this paper are derived from net changes of the geomagnetic field caused by the solar wind and magnetospheric processes. The daily baseline of the magnetic field, as well as the *S*_*q*_ current system, has been removed in the SuperMAG data. Short‐term variations in the magnetic field is also removed by a 3‐hr moving window for IMF reorientations and by a 30‐min moving window for ULF waves. For the cases of sudden solar wind pressure changes and substorms, only the net change in the magnetic field is used to derive the currents. The current system in our study is the net disturbance current system caused by the external process and does not include the background currents. Therefore, this disturbance current system is different from the system of the total currents.

## 4 Discussion

Ionospheric equivalent current systems caused by four different processes in the solar wind (solar wind pressure changes and IMF reorientations) or in the magnetosphere (substorms and ULF waves) are presented in this paper. Nishida (1968a, 1968b) and Huang (2019a) derived the global current distributions caused by IMF southward turnings (penetration electric fields). The current patterns presented in section 2.1 of this paper are consistent with the previous results. The current patterns related to penetration and shielding electric fields are included in this paper for the purpose of comparing the current patterns under different conditions. The global current patterns caused by sudden changes in the solar wind pressure, by magnetospheric substorms, and by ULF waves are all derived for the first time.

In this study, we use magnetometer data to derive ionospheric equivalent currents. Although this is an oversimplified method, Nishida (1968a, 1968b) and Kamide et al. (1976, 1981) showed that the method is pretty successful in deriving the global pattern of ionospheric currents. Motoba et al. (2003) and Huang (2019a) compared the ionospheric convection vectors based on magnetometer data with plasma velocity measurements by the Super Dual Auroral Radar Network (SuperDARN) and found reasonable agreement. The agreement provides justification for the method. In addition, the observed equivalent current distribution in the case of Pc5 pulsations is very similar to the model calculations of Imajo et al. (2017), and the agreement between the model and observations provides further justification for the method.

Our study focuses on the current distribution at middle and low latitudes. The ionospheric current distribution at high latitudes (above 60° MLat) is not very well defined in some cases, and the current vortex near dawn denote only the approximate location. Although there is some uncertainty in the current distribution at high latitudes, the double vortex current system caused by IMF reorientations and substorm expansion onset (those in Figures 21a and 21b) and the single vortex current system caused by solar wind pressure changes and ULF waves (those in Figures 21c and 21d), especially at middle and low latitudes, are very stable and reliable.

The current pattern in Figure 21a represents the one caused by IMF southward turning (penetration electric fields) and by onset of magnetospheric substorms. Following a southward turning of the IMF, the Region 1 FACs are enhanced and become stronger than the Region 2 FACs, and penetration electric fields occur at low latitudes (Nopper & Carovillano, 1978; Senior & Blanc, 1984). Similarly, following a substorm onset, the increase of the Region 1 FACs is larger than the increase of the Region 2 FACs (Forsyth et al., 2018), and penetration electric fields are observed at low latitudes (Huang, 2009, 2012).

In early studies of Obayashi and Nishida (1968) and Nishida (1968a, 1968b, 1971), the ionospheric current system related to penetration electric fields is termed DP 2 current system, characterized by a large vortex in the afternoon sector and a relatively small vortex in the morning sector, and the ionospheric current system caused by substorms is termed DP 1 current that can be a single vortex system or a double vortex system at high latitudes. Our results show that the ionospheric current systems related to penetration electric fields and substorm expansion phase, especially at middle and low latitudes, are nearly the same, and both are the DP 2 current system. The substorms analyzed in early studies may be isolated substorms, and the onset of isolated substorms is often triggered by an IMF northward turning or a solar wind pressure impulse (McPherron et al., 1986). The expansion phase of an isolated substorm triggered by a northward turning of the IMF often results in a westward electric field and counter electrojet in the dayside equatorial region (Kikuchi et al., 2000, 2001, 2003). In contrast, substorms during geomagnetic storms, such as the sawtooth events presented in this paper, often occur under continuous southward IMF, and the expansion phase of the sawtooth substorms without IMF northward turning causes an eastward electric field and electrojet (Huang, 2009, 2012). The difference in the ionospheric current system between sawtooth substorms and isolated substorms may be related to the IMF condition (IMF northward turning or continuous southward IMF) and the substorm‐induced electric fields.

The ionospheric equivalent current systems caused by solar wind pressure changes or magnetospheric ULF waves, as depicted in Figures 21c and 21d, are characterized by a single vortex at middle and low latitudes. A sudden enhancement or drop in the solar wind pressure first impacts on the dayside magnetopause and causes changes in the magnetopause currents. Fluctuations in the solar wind pressure can initiate surface wave mode via the Kelvin‐Helmholtz instability at the magnetopause boundary, and the surface waves can trigger field line resonances in the magnetosphere which appear as pulsations (Baumjohann & Treumann, 1997). Both the compression of the magnetosphere by a solar wind pressure impulse and the Kelvin‐Helmholtz instability generated by magnetosheath flows first occur on the dayside magnetopause and then affect the ionosphere, which may be the reason for that the ionospheric equivalent currents caused by a solar wind pressure discontinuity and by ULF waves have the same or similar patterns.

Motoba et al. (2003) studied the response of the magnetosphere‐ionosphere current system to a solar wind pressure oscillation and derived the dayside equivalent current vectors in the high‐latitude ionosphere. The disturbance current distributions showed the characteristics of a counterclockwise vortex near the peak of the solar wind pressure oscillations and a clockwise vortex near the valley of the solar wind pressure oscillations, respectively. In our study, the ionospheric current system at middle and low latitudes is characterized by a large counterclockwise vortex in response to a sudden enhancement in the solar wind pressure and by a large clockwise vortex in response to a sudden drop in the solar wind pressure, respectively. Our results are consistent with that of Motoba et al. (2003).

Another important finding of this study is that the disturbance current system is completely established within 1 min. This time scale for global ionospheric current system to establish or reconstruct is identified for the first time. It is also important to note that the current system established within 1 min remains unchanged for the next 20–30 min, indicating that a new quasi‐steady state of the ionospheric current distribution is reached within 1 min. As already mentioned earlier, the background magnetic fields and currents have been removed, the equivalent current system in this paper is the net change of the currents caused by the solar wind or magnetospheric processes and does not represent the total ionospheric currents. How significantly the pattern of the total currents is changed is a different topic and beyond the scope of this paper. If the background currents are assumed to be unchanged, the total currents (the background currents plus the net disturbance currents) will also reach a quasi‐steady state within 1 min. The results imply that the ionospheric current system (the system of the total currents) also takes only 1 min to change from one steady state to a new steady state.

The variation of the geomagnetic field in response to IMF reorientations, magnetospheric substorms, and ULF waves, is relatively slow, and the corresponding ionospheric current pattern evolves over a longer time scale. This longer time scale is determined by the driving process that changes gradually. If the external process is fast enough, the reconstruction of the ionospheric current system should be also very fast.

Because a steady state of the ionospheric current system can be established within 1 min, it implies that electromagnetic changes can be transmitted from the source region at high latitudes to all latitudes/longitudes very quickly, in a time scale of shorter than 1 min. One mechanism is the propagation of a zeroth‐order transverse magnetic mode in the Earth‐ionosphere waveguide (Kikuchi & Araki, 1979a, 1979b). In this model, electromagnetic fields can propagate from high to low latitudes instantaneously at the speed of light. This mechanism has been used to explain the occurrence of penetration electric fields at low latitudes in many subsequent studies. Kikuchi et al. (1996) found that polar electric fields penetrate to the equatorial ionosphere almost instantaneously within the time resolution of 25 s.

On the other hand, Chi et al. (2001) analyzed propagation of preliminary reverse impulse (PRI) of the geomagnetic field in response to a rapid increase of the solar wind pressure and suggested that low‐latitude PRI signals are the MHD waves. They also found implications that high‐latitude PRIs can be induced by the vortex of ionospheric currents at nearby latitudes, and the motion of the current vortex can affect the arrival time of high‐latitude PRIs. Recently, Tu and Song (2019) developed a numerical model to simulate transmission of the solar wind energy and momentum from the polar cap to equatorial latitudes through the fast magnetosonic waves, and the propagation time of the fast magnetosonic waves is a few seconds. After several oscillations, the ion velocity changes, as well as changes in the magnetic and thermal pressure, reach a steady state within a time scale of ~20 s.

Both the zeroth‐order transverse magnetic mode of Kikuchi and Araki (1979a, 1979b) and the fast magnetosonic waves of Tu and Song (2019) can explain the observations of ionospheric ion velocity and density perturbations, as well as electromagnetic perturbations, associated with penetration electric fields. The direction and strength of ionospheric currents are determined by the ion and electron flows and conductivity. Very fast establishment of a new steady state in ionospheric plasma density and velocity in response to a sudden change in the solar wind pressure results in the establishment of a new steady state of the current system within 1 min.

## 5 Conclusions

We have used magnetic field data measured by global magnetometer networks to derive ionospheric equivalent currents caused by IMF southward or northward turning (corresponding to the occurrence of penetration or shielding electric fields), a sudden increase or decrease in the solar wind pressure, onset of magnetospheric substorms during sawtooth events, and ULF waves. The background magnetic field is removed, and only the net changes in magnetic field are used to derive the equivalent currents. The derived equivalent disturbance current system represents the net current change caused by the solar wind or magnetospheric processes. The following conclusions are derived from 11 cases for the four processes.

The equivalent disturbance current system caused by an IMF southward turning (occurrence of penetration electric fields) or by the onset of a substorm is characterized by a large counterclockwise vortex covering the prenoon sector, afternoon sector, and evening sector and a relatively small clockwise vortex in the midnight‐dawn sector (or near dawn). The current pattern following an IMF northward turning (occurrence of shielding electric fields) is nearly the same as that for an IMF southward turning, but the polarity of the current vortices is reversed.

The equivalent disturbance current system caused by a sudden enhancement in the solar wind pressure or by ULF waves at wave peak is characterized by a large single counterclockwise vortex covering all longitudes/local times at middle and low latitudes and a very small clockwise vortex above ~60° MLat near dawn. The current pattern caused by a sudden drop in the solar wind pressure or by ULF waves at wave valley is nearly the same as that for a solar wind pressure enhancement and for a ULF wave peak, but the polarity of the current vortices is reversed.

The ionosphere takes only 1 min to establish the equivalent disturbance current system caused by a solar wind pressure enhancement, and the new steady‐state current system remains for 20–30 min without obvious deformation. If the ionospheric background current system is assumed to remain unchanged across the solar wind discontinuity, the system of the total currents (the background currents plus the net current changes) also takes only 1 min to reach a new steady state.

We propose the following scenario for the establishment of ionospheric equivalent disturbance current systems caused by a solar wind or magnetospheric process. An IMF southward turning, a sudden increase in the solar wind pressure, a substorm onset, or a ULF wave peak enhances the Region 1 FACs, depositing solar wind/magnetospheric energy and momentum to the high‐latitude ionosphere and causing enhanced convection electric fields. The electric fields at high latitudes, as well as energy and momentum, are transmitted, through the transverse magnetic mode or the fast magnetosonic wave mode, to low latitudes, generating penetration electric fields and related magnetic field changes. After several periods of the electromagnetic waves or the fast magnetosonic waves, the ion velocities in the global ionosphere, as well as electromagnetic fields, reach a new steady state, and a new steady‐state current system is established. Although the solar wind or magnetospheric processes can be very different, the ionospheric currents quickly reaches the quasi‐steady state within ~1 min. The single or double vortex current system depends on the distribution of the FACs in latitude and longitude/local time.
